# Plasma p-tau231 and p-tau217 as state markers of amyloid-β pathology in preclinical Alzheimer’s disease

**DOI:** 10.1038/s41591-022-01925-w

**Published:** 2022-08-11

**Authors:** Marta Milà-Alomà, Nicholas J. Ashton, Mahnaz Shekari, Gemma Salvadó, Paula Ortiz-Romero, Laia Montoliu-Gaya, Andrea L. Benedet, Thomas K. Karikari, Juan Lantero-Rodriguez, Eugeen Vanmechelen, Theresa A. Day, Armand González-Escalante, Gonzalo Sánchez-Benavides, Carolina Minguillon, Karine Fauria, José Luis Molinuevo, Jeffrey L. Dage, Henrik Zetterberg, Juan Domingo Gispert, Marc Suárez-Calvet, Kaj Blennow

**Affiliations:** 1 Barcelonaβeta Brain Research Center (BBRC), Pasqual Maragall Foundation, Barcelona, Spain; 2 IMIM (Hospital del Mar Medical Research Institute), Barcelona, Spain; 3 Centro de Investigación Biomédica en Red de Fragilidad y Envejecimiento Saludable, Instituto de Salud Carlos III, Madrid, Spain; 4 Universitat Pompeu Fabra, Barcelona, Spain; 5 Department of Psychiatry and Neurochemistry, Institute of Neuroscience and Physiology, University of Gothenburg, Mölndal, Sweden; 6 Wallenberg Centre for Molecular and Translational Medicine, University of Gothenburg, Gothenburg, Sweden; 7 King’s College London, Institute of Psychiatry, Psychology & Neuroscience, Maurice Wohl Clinical Neuroscience Institute, London, UK; 8 NIHR Biomedical Research Centre for Mental Health & Biomedical Research Unit for Dementia at South London & Maudsley NHS Foundation, London, UK; 9 Translational Neuroimaging Laboratory, McGill Centre for Studies in Aging, McGill University, Montreal, Quebec Canada; 10 Department of Psychiatry, University of Pittsburgh, Pittsburgh, PA USA; 11 ADx NeuroSciences NV, Ghent, Belgium; 12 Lilly Research Laboratories, Eli Lilly and Company, Indianapolis, IN USA; 13 Stark Neurosciences Research Institute, Indiana University School of Medicine, Indianapolis, IN USA; 14 Clinical Neurochemistry Laboratory, Sahlgrenska University Hospital, Mölndal, Sweden; 15 Department of Neurodegenerative Disease, UCL Institute of Neurology, London, UK; 16 UK Dementia Research Institute at UCL, London, UK; 17 Centro de Investigación Biomédica en Red Bioingeniería, Biomateriales y Nanomedicina, Madrid, Spain; 18 Servei de Neurologia, Hospital del Mar, Barcelona, Spain

**Keywords:** Predictive markers, Alzheimer's disease

## Abstract

Blood biomarkers indicating elevated amyloid-β (Aβ) pathology in preclinical Alzheimer’s disease are needed to facilitate the initial screening process of participants in disease-modifying trials. Previous biofluid data suggest that phosphorylated tau231 (p-tau231) could indicate incipient Aβ pathology, but a comprehensive comparison with other putative blood biomarkers is lacking. In the ALFA+ cohort, all tested plasma biomarkers (p-tau181, p-tau217, p-tau231, GFAP, NfL and Aβ42/40) were significantly changed in preclinical Alzheimer’s disease. However, plasma p-tau231 reached abnormal levels with the lowest Aβ burden. Plasma p-tau231 and p-tau217 had the strongest association with Aβ positron emission tomography (PET) retention in early accumulating regions and associated with longitudinal increases in Aβ PET uptake in individuals without overt Aβ pathology at baseline. In summary, plasma p-tau231 and p-tau217 better capture the earliest cerebral Aβ changes, before overt Aβ plaque pathology is present, and are promising blood biomarkers to enrich a preclinical population for Alzheimer’s disease clinical trials.

## Abstract (web summary)

A comprehensive comparison of Alzheimer’s disease blood biomarkers in cognitively unimpaired individuals reveals that plasma p-tau231 and p-tau217 capture very early Aβ changes, showing promise as markers to enrich a preclinical population for Alzheimer’s disease clinical trials

## Main

Blood biomarkers that accurately indicate Alzheimer’s disease (AD) pathophysiology now offer a realistic, cost-effective and noninvasive assessment that will aid the diagnostic process in primary and secondary care. Plasma measures of phosphorylated tau at Thr181 (p-tau181), Thr217 (p-tau217) and Thr231 (p-tau231) have high diagnostic accuracy in differentiating AD from other neurodegenerative disorders in clinical studies^1–3^, which are validated by postmortem neuropathological studies^1,2,4^. In some instances^1^, the performance of plasma p-tau biomarkers is comparable or only marginally inferior to established cerebrospinal fluid (CSF) or PET examinations of Aβ and tau pathologies, but with the advantage of greater availability and tolerability for both clinicians and patients.

There is often discordance between the clinical diagnosis of AD and neuropathological findings. Thus, a noninvasive indicator that can improve the confidence in such a decision during life is paramount. This biological indication is also critically important in the preclinical stage of the AD continuum^5^ (hereafter, preclinical AD), where cerebral Aβ pathology is accumulating but individuals are cognitively unimpaired (CU). However, it is not yet clear how blood biomarkers will inform on the preclinical evaluation of AD. As anti-Aβ therapeutic trials move toward the assessments in the preclinical phase, a cost-effective tool is needed to reduce the number of lumbar punctures and PET scans in the recruitment process. Moreover, a blood biomarker would reduce recruitment time and increase the level of participation from more diverse populations that better represent the global aging population. Indeed, blood measures of p-tau181, p-tau217, p-tau231, glial fibrillary acid protein (GFAP), neurofilament light (NfL) and Aβ42/40 have been shown to change in preclinical AD and can discriminate this state from CU individuals with non-AD pathological changes^1,3,6–13^. Yet, our previous results in CSF and, more recently, plasma suggest that the earliest change in the AD continuum may be better characterized by p-tau231. CSF p-tau231 showed the earliest change in association with Aβ pathology in the AD brain^9,14^. Subsequently, the first blood analysis of p-tau231 (ref. ^2^) demonstrated earlier increases than plasma p-tau181 in a small set of participants.

Amid these promising results, a direct comparison of the main plasma biomarkers in a large number of individuals with preclinical AD is still needed. This will also determine the threshold of Aβ burden at which these biomarkers change in blood. Therefore, the main aim of our study is to investigate the main p-tau blood biomarkers for AD (p-tau181, p-tau217, p-tau231) together with the other relevant AD-related blood biomarkers (GFAP, NfL, Aβ42/40) in preclinical AD and compare their capacity to indicate Aβ pathology in CU individuals. For these purposes, we leverage the unique characteristics of the ALFA+ cohort^15,16^, which is composed of 397 CU middle-aged individuals (61.1 ± 4.67 years), 135 (34.0%) of whom are Aβ positive as defined by CSF Aβ42/40, a state marker reflecting the balance between production and clearance of Aβ^17^, and hence fall into preclinical AD (Supplementary Table 1). In addition, we used Aβ PET as a stage marker using two cut-offs. An early cut-off of Centiloids ≥12 (53 (15.6%) participants) is used to detect early Aβ aggregation in CU individuals, when Aβ pathology may be emerging^18,19^, and a later cut-off of Centiloids ≥30 (26 (7.7%) participants), reflecting more established Aβ plaque pathology^18–20^.

**Fig. 1:**
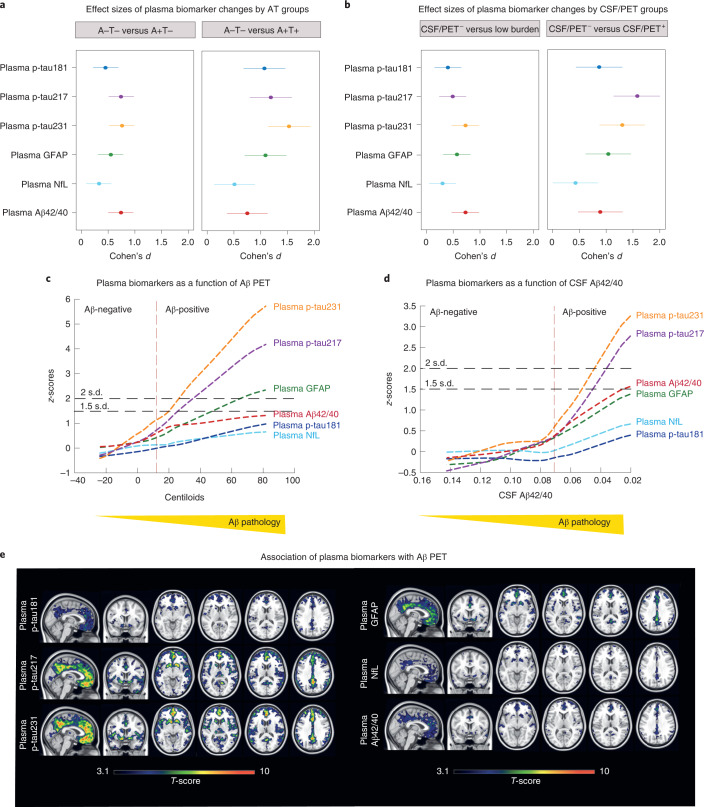
Plasma biomarkers and Aβ pathology. **a**,**b**, Effect sizes of plasma biomarker levels change by AT groups (**a**; *n* = 397; *n* = 249 A−T−, *n* = 104 A+T−, *n* = 31 A+T+, *n* = 13 A−T+) and by CSF/PET groups (**b**; *n* = 339; *n* = 224 CSF/PET Aβ negative, *n* = 89 low burden, *n* = 26 CSF/PET Aβ positive). Individuals with a low burden of Aβ pathology were defined as CSF Aβ42/40 <0.071 and Aβ PET <30 Centiloids. The effect size of group differences was estimated by calculating Cohen’s *d*, in which the dependent variable was the residual of log(transformed) plasma biomarkers regressed on age and sex. The error bars denote the 95% CIs. **c**,**d**, The graphs represent the *z*-score changes of each plasma biomarker using the mean and the s.d. of that plasma biomarker in the group of participants with CSF Aβ42/40 >0.1 as a reference. The resulting *z*-scores are shown as a function of Aβ PET Centiloids (**c**) or CSF Aβ42/40 (**d**) using a robust local weighted regression method. The vertical dashed lines depict the Aβ PET 12 Centiloids (**c**) and CSF Aβ42/40 positivity cut-off (**d**). The horizontal dashed lines depict the abnormality threshold held at 1.5 and 2 s.d. above the mean. The horizontal axis direction of CSF Aβ42/40 (**d**) was inverted. **e**, Association of plasma biomarkers with Aβ PET at the voxel level. Associations were tested using voxel-wise, univariate, independent, linear regression models with age and sex as covariates. All plasma biomarkers showed a significant association with Aβ deposition in orbitofrontal and precuneus. These associations were stronger with plasma p-tau231 and p-tau217 and also extended to the insula and striatum. Statistical significance was set at *P* < 0.001 uncorrected for multiple comparisons with a cluster size of *k* > 100 voxels. All tests were one sided but contrasts in both directions were tested. No significant associations were found in the opposite direction. Statistical maps were resliced to 0.5 mm^3^ (cubic) for visualization purposes.

**Extended Data Fig. 1:**
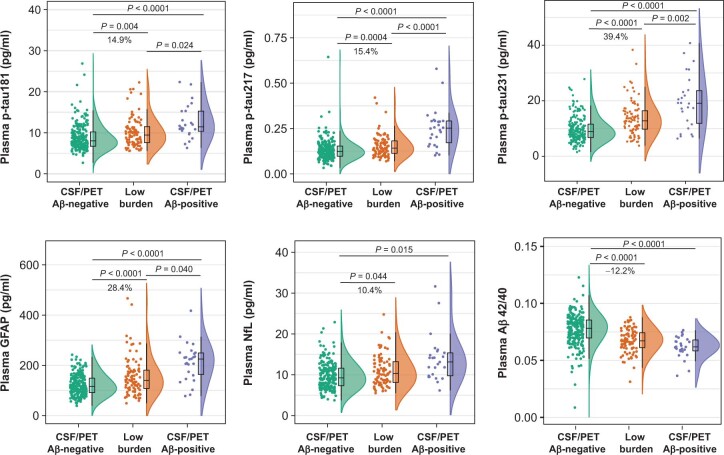
Plasma biomarkers by CSF/PET Aβ groups. Violin plots comparing plasma biomarkers between CSF/PET Aβ groups (*n* = 339; *n* = 224 CSF/PET Aβ-negative, *n* = 89 Low burden, *n* = 26 CSF/PET Aβ-positive). Individuals with a low burden of Aβ pathology were defined as CSF Aβ42/40 < 0.071 and Aβ PET Centiloid < 30. The box plots depict the median (horizontal bar), interquartile range (IQR, hinges), and 1.5 × IQR (whiskers). Group comparisons were computed with a one-way ANCOVA adjusting for age and sex, followed by Tukey-corrected *post hoc* pairwise comparisons. The percentage (%) of change in mean levels of plasma biomarkers in the low burden group compared to the CSF/PET Aβ-negative group is shown.

We first found that all plasma biomarkers were significantly changed in CU individuals who were Aβ positive (A+, as defined by CSF Aβ42/40 <0.071) but still tau negative (T−, as defined by CSF Mid(M)-p-tau181 ≤24 pg ml^−1^)^15^ (Fig. 1a and Supplementary Fig. 1). Plasma p-tau231, p-tau217 and Aβ42/40 showed the highest degree of change in this group (*P* < 0.0001; Cohen’s *d* = 0.76 for plasma p-tau231 and *d* = 0.74 for plasma p-tau217 and Aβ42/40), and were followed by GFAP (*P* < 0.0001; Cohen’s *d* = 0.55), p-tau181 (*P* = 0.001; Cohen’s *d* = 0.45) and NfL (*P* = 0.031; Cohen’s *d* = 0.33). All plasma biomarkers were also changed in the group of individuals with a low burden of Aβ pathology, namely those individuals who had abnormal CSF Aβ42/40 levels (and hence changes in soluble Aβ have started) but an Aβ PET <30 Centiloids (hence, not yet established Aβ plaque pathology) (Fig. 1b and Extended Data Fig. 1). Plasma p-tau231 and Aβ42/40 showed the highest degree of change in this group (*P* < 0.0001; Cohen’s *d* = 0.73), followed by GFAP (*P* < 0.0001; Cohen’s *d* = 0.57), p-tau217 (*P* = 0.0004; Cohen’s *d* = 0.49), p-tau181 (*P* = 0.004; Cohen’s *d* = 0.40) and NfL (*P* = 0.044; Cohen’s *d* = 0.30). To confirm the early changes of plasma biomarkers in the AD continuum, we applied a robust local weighted regression method to model their trajectories across preclinical AD using Aβ PET (Fig. 1c) and CSF Aβ42/40 (Fig. 1d) as proxies for the disease progression^17^. For Aβ PET, we observed that plasma p-tau231 was the first blood biomarker to surpass the two *z*-score levels (used here as a definition of abnormality; Fig. 1c) at a corresponding Aβ PET of 26.4 Centiloids, followed by plasma p-tau217 (35.4 Centiloids) and plasma GFAP (65.5 Centiloids). Plasma p-tau181, NfL and Aβ42/40 did not reach this abnormality threshold. Using CSF Aβ42/40 as a proxy of disease progression, plasma p-tau231 and plasma p-tau217 showed a parallel and steep increase and were the only plasma biomarkers to surpass the two *z*-score threshold (Fig. 1d). We also investigated the voxel-wise associations between Aβ PET and each of the plasma biomarkers (Fig. 1e), and found that plasma p-tau231 and p-tau217 were the plasma biomarkers that had the strongest association with Aβ PET in areas known to show early Aβ accumulation, namely the orbitofrontal areas, anterior and posterior cingulate gyri, insula and precuneus. In contrast, the other biomarkers had weaker and less widespread associations across the brain with, in particular, less involvement of the insula (Fig. 1e). Correlations between plasma and CSF biomarkers are shown in Supplementary Figs. 2 and 3.

**Extended Data Fig. 2:**
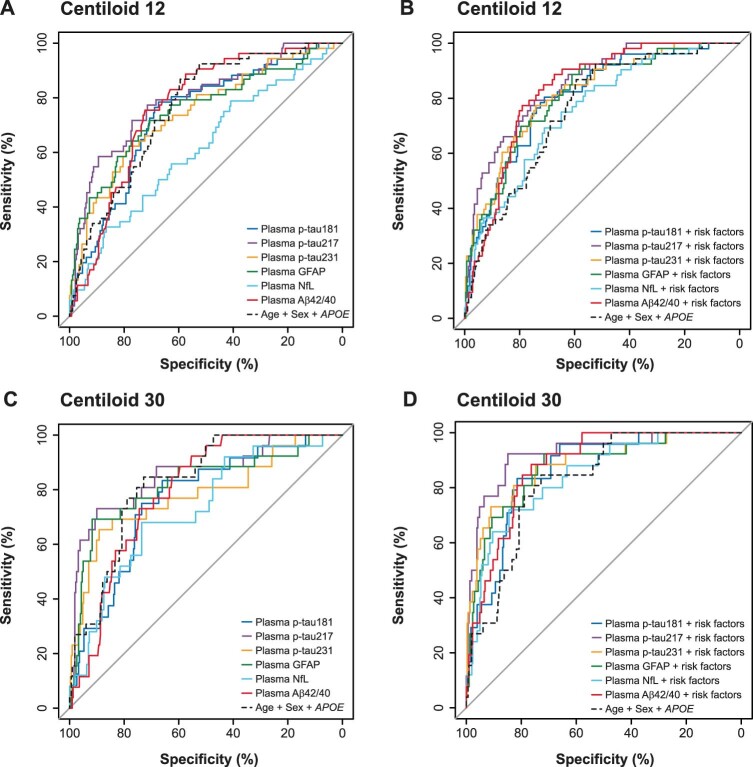
ROC curves for the discrimination of Aβ PET Centiloid 12 or 30. ROC analysis was performed to test the accuracy of plasma biomarkers (A), and plasma biomarkers in combination with a base risk factors model (age, sex and *APOE* ε4 status) (B), to discriminate participants with Aβ PET Centiloid ≥ 12 from those with Aβ PET Centiloid < 12. The same analyses were performed on both plasma biomarkers alone (C) or in combination with a base risk factors model (D) to discriminate participants with Aβ PET Centiloid ≥ 30 from those with Aβ PET Centiloid < 30.

We next examined the accuracy of the different plasma biomarkers to detect Aβ pathology, as measured by Aβ PET or CSF Aβ42/40, in CU individuals. We performed receiver operating characteristic (ROC) analyses and the resulting areas under the curve (AUCs) for each plasma biomarker, and their combinations with risk factors (sex, age and *APOE* ε4 status) were compared with a base model including only AD risk factors using DeLong’s test. When it comes to the discrimination of early Aβ pathology (Aβ PET burden ≥12 Centiloids), none of the plasma biomarkers alone significantly improved the base risk factors model. Yet, the combination of plasma p-tau181, p-tau217, p-tau231 or Aβ42/40 with the base risk factors model outperformed the base risk factors model alone, but plasma p-tau181 and p-tau231 did not survive correction for multiple comparisons (Extended Data Fig. 2 and Supplementary Table 2). When it comes to established Aβ pathology (Aβ PET burden ≥30 Centiloids), the combination of the base risk factors model with plasma p-tau217, Aβ42/40 or p-tau231 outperformed the base risk factors model alone, but plasma p-tau231 did not survive multiple comparison correction (Extended Data Fig. 2 and Supplementary Table 2).

**Extended Data Fig. 3:**
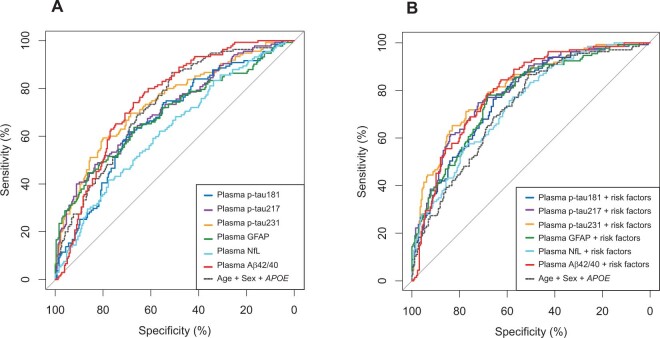
ROC curves for the discrimination of Aβ status (CSF Aβ42/40). ROC analysis was performed to test the accuracy of plasma biomarkers (A), and plasma biomarkers in combination with a base risk factors model (age, sex and *APOE* ε4 status) (B), to discriminate between Aβ status as defined by CSF Aβ42/40.

When assessing Aβ status based on CSF Aβ42/40 (Table 1 and Extended Data Fig. 3), which assesses soluble Aβ and changes earlier than Aβ PET^21^, the highest AUCs were reached by plasma Aβ42/40 (AUC = 0.750 (95% confidence interval (CI) = 0.702–0.798)) and p-tau231 (AUC = 0.740 (95% CI = 0.688–0.793)). DeLong’s test revealed that plasma biomarkers performed similarly well, with only plasma p-tau231 and Aβ42/40 being significantly better than plasma NfL. In line with Aβ PET results, the performance of plasma biomarkers did not improve that of the base risk factors model to indicate Aβ pathology as defined by decreased CSF Aβ42/40 (AUC = 0.729 (95% CI = 0.678–0.779)). However, the addition of any plasma biomarker, except for NfL, to the base risk factors model significantly increased its performance. In particular, the highest AUCs were for risk factors combined with plasma p-tau231 (AUC = 0.810 (95% CI = 0.766–0.854)), plasma Aβ42/40 (AUC = 0.798 (95% CI = 0.754–0.843)) and plasma p-tau217 (AUC = 0.797 (95% CI = 0.751–0.842)) (Table 1 and Extended Data Fig. 3). We next assessed whether the accuracy of the plasma biomarkers differs with age, because that may be relevant to better understand the plasma biomarker changes across the continuum and also to define inclusion criteria in prevention clinical trials. We performed the ROC analyses separately in a younger (≤65 years; *n* = 309) and an older (>65 years; *n* = 88) age group (Table 1 and Supplementary Table 3). In the younger age group, the combination of the base risk factors model with plasma p-tau181, p-tau217, p-tau231, GFAP or Aβ42/40 was significantly better than the base risk factors model (Table 1), whereas in the older group only plasma p-tau217 and p-tau231 (the latter at nominal level) were significantly better than the base risk factors model (Table 1). We repeated the analyses stratifying by the median age of the sample (61.8 years). In the CU individuals aged ≤61.8 years, the combination of the risk factors model with plasma p-tau231 (AUC = 0.847 (95% CI = 0.791–0.903)) or plasma Aβ42/40 (AUC = 0.828 (95% CI = 0.767–0.888)) was the only model that significantly outperformed the base risk factors model (Supplementary Table 4).

**Table 1:** ROC analyses to discriminate Aβ status (defined by CSF Aβ42/40)

	All (*n* = 397)			Younger group (≤65 yo; *n* = 309)			Older group (>65 yo; *n* = 88)		
	AUC (95% CI)	*P* value	*P*_adj_ value	AUC (95% CI)	*P* value	*P*_adj_ value	AUC (95% CI)	*P* value	*P*_adj_ value
Base risk factors model (age + sex + *APOE* ε4)	0.729 (0.678–0.779)			0.710 (0.649–0.770)			0.730 (0.625–0.835)		
versus plasma p-tau181	0.672 (0.616–0.729)	0.12	0.36	0.667 (0.599–0.734)	0.36	0.43	0.637 (0.518–0.755)^f^	0.30	0.59
versus plasma p-tau217	0.711 (0.656–0.765)	0.62	0.74	0.666 (0.597– 0.734)	0.35	0.43	0.834 (0.753–0.915)^g^	0.11	0.37
versus plasma p-tau231	0.740 (0.688–0.793)^a^	0.78	0.78	0.736 (0.674–0.798)^a^	0.59	0.59	0.753 (0.652–0.855)	0.77	0.92
versus plasma GFAP	0.691 (0.632–0.749)	0.26	0.51	0.661 (0.589–0.732)	0.21	0.43	0.716 (0.605–0.827)	0.92	0.92
versus plasma NfL	0.623 (0.565–0.682^)b^	0.007^*^	0.042^*^	0.600 (0.529–0.670)^e^	0.023^*^	0.14	0.581 (0.457–0.704)	0.12	0.37
versus plasma Aβ42/40	0.750 (0.702–0.798)	0.53	0.74	0.754 (0.698–0.810)	0.29	0.43	0.709 (0.597–0.820)	0.79	0.92
Base risk factors model (age + sex + *APOE* ε4)	0.729 (0.678–0.779)			0.710 (0.649–0.770)			0.730 (0.625–0.835)		
versus plasma p-tau181 + risk factors	0.770 (0.722–0.817)	0.014^*^	0.021^*^	0.770 (0.715–0.826)	0.011^*^	0.021^*^	0.749 (0.647–0.850)^h^	0.52	0.62
versus plasma p-tau217 + risk factors	0.797 (0.751–0.842)^c^	0.0005^*^	0.0010^*^	0.765 (0.709–0.821)	0.019^*^	0.029^*^	0.887 (0.823–0.952)^c^	0.001^*^	0.008^*^
versus plasma p-tau231 + risk factors	0.810 (0.766–0.854)^c^	0.0002^*^	0.0006^*^	0.811 (0.761–0.860)	0.0002^*^	0.0006^*^	0.829 (0.743–0.915)	0.044^*^	0.13
versus plasma GFAP + risk factors	0.773 (0.724–0.821)	0.020^*^	0.024^*^	0.764 (0.708–0.821)	0.028^*^	0.033^*^	0.793 (0.697–0.888)	0.12	0.25
versus plasma NfL + risk factors	0.743 (0.693–0.793^)d^	0.18	0.18	0.742 (0.684–0.800)	0.13	0.13	0.728 (0.622–0.834)	0.76	0.76
versus plasma Aβ42/40 + risk factors	0.798 (0.754–0.843)	<0.0001^*^	0.0003^*^	0.803 (0.753–0.854)	<0.0001^*^	0.0003^*^	0.776 (0.677–0.875)	0.17	0.26

*APOE*, apolipoprotein E; yo, years old.

ROC analyses for the discrimination between Aβ-positive (A+) and Aβ-negative (A−) individuals, as defined by the CSF Aβ42/40 ratio (cut-off for positivity: CSF Aβ42/40 <0.071). Participants were stratified by age into two groups: a younger (age ≤65 years) and an older (age >65 years) group. Demographic characteristics of these groups are shown in Supplementary Table 3. In a first model, we compared each plasma biomarker with the base risk factor model (age, sex and *APOE* ε4 status), and in a second model we combined each plasma biomarker with the risk factors. *P* values refer to the comparisons with the base risk factor model. Biomarker models were also compared between them. AUC differences were tested using a two-sided DeLong’s test followed by FDR multiple comparison correction. Adjusted and nonadjusted *P* values are shown. *Significant values compared with the base risk factor model.

^a^*P* < 0.05 versus plasma NfL.

^b^*P* < 0.01 versus plasma Aβ42/40.

^c^*P* < 0.05 versus plasma NfL + risk factors.

^d^*P* < 0.05 versus plasma Aβ42/40 + risk factors.

^e^*P* < 0.05 versus plasma Aβ42/40.

^f^*P* < 0.05 versus plasma p-tau217.

^g^*P* < 0.01 versus plasma NfL.

^h^*P* < 0.05 versus plasma p-tau217 + risk factors.

We calculated cut-off points for the discrimination of Aβ status (as defined by CSF Aβ42/40) for each plasma biomarker using Youden’s index or setting sensitivity at 85% (Supplementary Table 5). After setting sensitivity at 85%, all combinations of plasma biomarkers with the base risk factors model, except for plasma NfL, reached a specificity >50%. In addition, we also performed these analyses with Aβ PET as a marker of Aβ burden (Supplementary Table 6).

**Extended Data Fig. 4:**
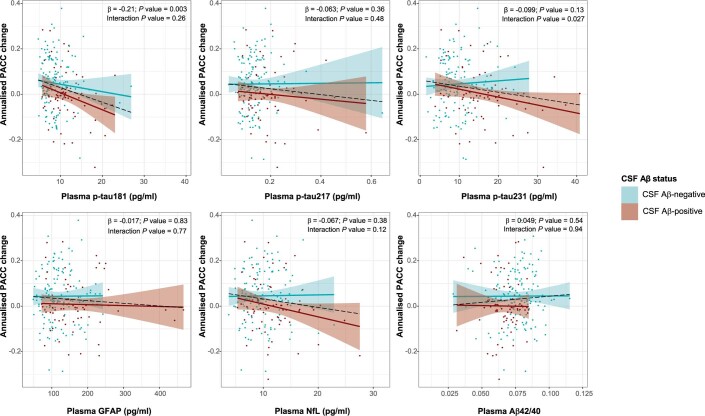
Association of plasma biomarkers with longitudinal change in cognition by Aβ status (CSF Aβ42/40). Scatter plots representing the associations of each of the plasma biomarkers with annualized change in PACC scores. Each point depicts the value of the plasma biomarker of an individual and the solid lines indicate the regression line for each of the groups. The dashed line indicates the regression line of the whole sample. The error bands denote the 95% CIs. The standardized regression coefficients (β) and *P* values are shown and were computed using a linear regression with the annualized change in PACC scores as the dependent variable, adjusting by age, sex and years of education. All tests were two-sided. Annualized change in PACC scores was computed as the subtraction of PACC scores at visit 2 minus those at visit 1 divided by the time difference between the two visits in years. At the nominal level, there was a significant interaction between CSF Aβ status (as defined by CSF Aβ42/40) and plasma p-tau231. Thus, we performed a stratified analysis by CSF Aβ status for this biomarker, and we found a significant association of plasma p-tau231 with PACC score longitudinal changes in the CSF Aβ-positive group (β = –0.27, *P* = 0.023) but not in the Aβ-negative group (β = 0.054; *P* = 0.51). See Supplementary Table 8 for the detailed analyses, including FDR correction for multiple testing.

**Extended Data Fig. 5:**
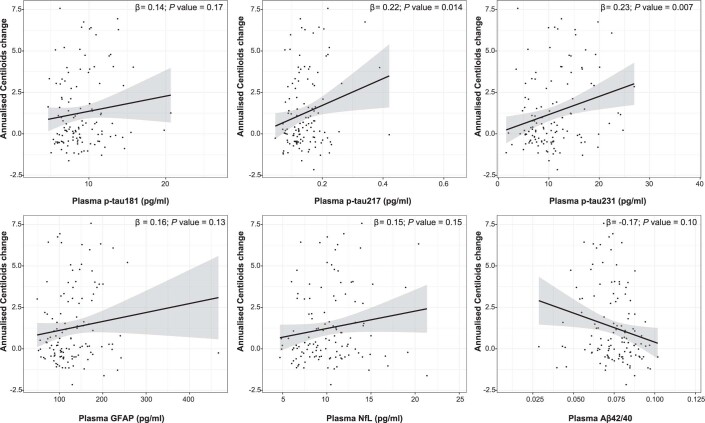
Association of plasma biomarkers with longitudinal change in Aβ deposition in individuals with Aβ PET Centiloids < 30. In order to assess whether plasma biomarkers are associated with longitudinal Aβ aggregation in the earliest stages of the Alzheimer’s *continuum*, we conducted a sensitivity analysis in those individuals with Aβ PET Centiloid < 30. Plasma p-tau231 and p-tau217 were significantly associated with Aβ PET Centiloid increases. Scatter plots represent the associations of each of the plasma biomarkers with annualized change in Aβ PET Centiloids in individuals with Aβ PET Centiloids < 30. Each point depicts the value of the plasma biomarker of an individual and the solid lines indicate the regression line. The error bands denote the 95% CIs. The standardized regression coefficients (β) and *P* values are shown and were computed using a linear regression with the annualized change in Aβ PET Centiloids as the dependent variable, adjusting by age and sex. All tests were two-sided. Annualized change in Aβ PET Centiloids was computed as the subtraction of Centiloid values at visit 2 minus those at visit 1 divided by the time difference between the two visits in years. See Supplementary Table 10 for the detailed analyses, including FDR correction for multiple testing.

We investigated whether baseline plasma biomarkers were associated with cognitive changes after 3 years of follow-up in a subset of participants with available data (*n* = 214; Supplementary Table 7). In the whole group, plasma p-tau181 was significantly associated with cognitive decline (as measured by the preclinical Alzheimer cognitive composite (PACC); *P* = 0.020), whereas p-tau231 was the only plasma biomarker with a significant interaction with Aβ status (as defined by CSF Aβ42/40) at the nominal level (*P* = 0.027) (Supplementary Table 8 and Extended Data Fig. 4). After stratification by CSF Aβ status, plasma p-tau231 was associated with cognitive decline in the Aβ-positive group (*P* = 0.023). Finally, we assessed whether baseline plasma biomarkers were associated with Aβ PET Centiloid changes after 3 years of follow-up (*n* = 145; Supplementary Table 7). All plasma biomarkers were associated with an increase in Aβ PET Centiloids but only the interaction between plasma p-tau231 and Aβ status was nominally significant (*P* = 0.015) (Supplementary Table 9 and Supplementary Fig. 4). We performed sensitivity analysis in those participants with <30 Centiloids, and hence no established Aβ pathology at baseline, and only plasma p-tau231 and p-tau217 were significantly associated with Aβ PET Centiloid increases at follow-up (*P* = 0.041, both; Supplementary Table 10 and Extended Data Fig. 5).

In summary, we demonstrate that plasma biomarkers change in the preclinical stage of the AD continuum but with differences among them. Several pieces of evidence consistently support plasma p-tau231 and p-tau217 being biomarkers indicating very early Aβ changes. First, plasma p-tau231 reaches abnormal levels at only 26.4 Centiloids and plasma p-tau217 at 35.4 Centiloids. Second, both plasma p-tau231 and p-tau217 had the strongest association with Aβ PET uptake in brain areas with known early Aβ deposition. Third, we show that, in individuals who have not yet established Aβ pathology at baseline (Aβ PET <30 Centiloids), plasma p-tau231 and p-tau217 are associated with longitudinal increases in Aβ PET uptake. Of note, plasma p-tau231 was associated with cognitive decline in Aβ-positive CU individuals. Moreover, plasma p-tau231, together with plasma Aβ42/40, has the largest change in the group with a low Aβ burden, and they both, in combination with age, sex and *APOE* ε4 status, also show the higher AUC to indicate Aβ pathology in the younger CU individuals, when Aβ pathology presumably starts. Conversely, plasma p-tau217, p-tau231, GFAP and Aβ42/40 are all adequate to detect established Aβ pathology (as measured by Aβ PET, a stage biomarker).

Some study limitations should be noted. First, different platforms have been used to measure the plasma biomarkers and the contribution of assay platform in regard to diagnostic accuracy remains unclear. Second, ALFA+ includes participants with a higher risk for AD by design (high prevalence of *APOE* ε4 carriership and Aβ positivity) and, therefore, it does not represent normal aging in the general population.

Amid the recent developments in anti-Aβ therapies and the increasing awareness of treating AD as early as possible, the use of plasma biomarkers—particularly p-tau231 and p-tau217—will facilitate the recruitment of participants in clinical trials at this early stage of the disease, but the choice of the plasma biomarker may differ depending on its goal. Plasma p-tau231 may be more suited to trials in middle-aged individuals with changes in soluble Aβ but subthreshold levels of Aβ pathology in PET, whereas other plasma biomarkers also have a satisfactory performance in older individuals and/or in the presence of established Aβ PET pathology.

## Methods

### Participant characteristics

The present study was performed in the ALFA+ cohort, a nested longitudinal study from the ALFA (for ALzheimer’s and FAmilies) study^16^. The ALFA study includes 2,743 middle-aged, CU individuals, with a high proportion of AD patients’ offspring (47.4%) and *APOE* ε4 carriers (34.7%).

The ALFA+ study includes 450 participants who were invited to participate based on their specific AD risk profile, determined by an algorithm in which participants’ AD parental history and *APOE* status, verbal episodic memory score and CAIDE score were taken into consideration^16^. A detailed phenotyping was performed in ALFA+ participants, including a lumbar puncture for the measurement of CSF biomarkers and imaging (magnetic resonance imaging (MRI) and positron emission tomography (PET)) biomarker acquisition. ALFA+ inclusion criteria were: (1) individuals who had previously participated in the ALFA study; (2) age between 45 and 65 years at the moment of inclusion in ALFA; and (3) long-term commitment to the study: inclusion and follow-up visits and agreement to undergo all tests and study procedures (MRI, PET and lumbar puncture). ALFA+ exclusion criteria were: (1) cognitive impairment (Clinical Dementia Rating (CDR) >0, Mini-Mental State Examination (MMSE) <27 or semantic fluency <12); (2) any systemic illness or unstable medical condition that could lead to difficulty complying with the protocol; (3) any contraindication to any test or procedure; and (4) a family history of monogenic AD. In the present study, we included 397 individuals with available baseline CSF and plasma biomarker measurements, of whom 339 also had available baseline Aβ PET. A subset of participants had longitudinal cognitive (*n* = 214) and Aβ PET (*n* = 145) data (follow-up of 3 years).

We classified ALFA+ participants as Aβ positive (A+) if CSF Aβ42/40 <0.071 and tau positive (T+) if CSF Mid(M)-p-tau181 >24 pg ml^−1^ (ref. ^15^) We further classified participants according to their CSF/PET Aβ status. The group with a low burden of Aβ pathology was defined as CSF Aβ42/40 <0.071 and Aβ PET Centiloids <30 and was compared with CSF/PET Aβ negative (CSF Aβ42/40 ≥0.071 and Aβ PET Centiloids <30) and CSF/PET Aβ positive (CSF Aβ42/40 <0.071 and Aβ PET Centiloids ≥30).

In addition, we used Aβ PET as a stage biomarker with two cut-points, an early cut-off (12 Centiloids), where pathology may be emerging, and a later cut-point (30 Centiloids), reflecting established Aβ pathology. The 12-Centiloid threshold is the optimal cut-off validated in neuropathology to detect CERAD moderate-to-frequent, neuritic plaque scores^19^, early detection of Aβ abnormalities by PET^22^ and agreement against CSF AD biomarkers^18^. Our choice of 30 Centiloids as a later cut-off was based on our previous findings that it has the best agreement with the CSF t-tau/Aβ42 ratio in pooled data of ALFA+ and AD neuroimaging (ADNI) cohort biomarkers^18^. This is also in line with the findings that showed that 26 Centiloids is an optimal cut-off in agreement with visual reads, which has been validated against CERAD pathology^23^, and with the 35.7 Centiloid cut-off for established Aβ abnormalities in PET described by Bullic et al.^22^. Moreover, the range from 12 Centiloids to 30 Centiloids has been proposed to reflect the ‘gray zone’ of Aβ deposition^24^.

The ALFA+ study (ALFA-FPM-0311) was approved by the independent ethics committee ‘Parc de Salut Mar’, Barcelona, and registered at Clinicaltrials.gov (identifier: NCT02485730). All participating subjects signed the study’s informed consent form which had also been approved by the independent ethics committee ‘Parc de Salut Mar’, Barcelona.

### Sample collection and biomarker measurements

The CSF sample collection and processing followed standard procedures^25^ and have been described previously^15^. In short, participants fasted for at least 8 h and a lumbar puncture was performed at the intervertebral space L3–L4, L4–L5 or L5–S1 using a standard needle. CSF was collected into a 15-ml sterile polypropylene sterile tube (Sarstedt, catalog no. 62.554.502), aliquoted in volumes of 0.5 ml into sterile poly(propylene) tubes (0.5-ml Screw Cap Micro Tube Conical Bottom, catalog no. 72.730.005) and immediately frozen at −80 °C.

The blood sample collection and processing procedure have been described previously^9^. Blood samples were obtained on the same day as the lumbar puncture in fasting conditions. Whole blood was drawn with a 20G or 21G needle gauge into a 10-ml EDTA tube (BD Hemogard, 10 ml, K2EDTA, catalog no. 367525). Tubes were gently inverted 5–10 times and centrifuged at 2,000*g* for 10 min at 4 °C. The supernatant was aliquoted in volumes of 0.5 ml into sterile poly(propylene) tubes (Sarstedt Screw Cap Micro Tube, 0.5 ml, PP, ref. no. 72.730.105) and immediately frozen at −80 °C. The samples were processed at room temperature. The time between collection and freezing of both CSF and plasma samples was <30 min.

All CSF and plasma biomarkers, except for CSF and plasma p-tau217, were analyzed at the Clinical Neurochemistry Laboratory at the University of Gothenburg, Sweden. Measurements of CSF p-tau181 and GFAP (Simoa platform) and CSF p-tau231 (ELISA) have been described previously^3,12^. CSF Aβ40, Aβ42 and NfL were measured with the exploratory NTK robust immunoassays (Roche Diagnostic International Ltd) on a cobas e 411 analyzer or cobas e 601 module. CSF M-p-tau181 and M-t-tau (both corresponding to the mid-region (M) domain of tau protein) were measured using the electrochemiluminescence Elecsys Phospho-Tau (181P) CSF and total-tau CSF immunoassays, respectively, on a fully automated cobas e 601 module (Roche Diagnostics International Ltd)^9^.

Plasma GFAP and NfL were quantified with GFAP Discovery (no. 102336) and NF-light Advantage (no. 103186) commercial kits, respectively. Plasma Aβ42/40 was measured with the commercial Neurology 4-Plex E Advantage Kit (no. 103670). New plasma p-tau181 was measured using an in-house Simoa assay developed at the University of Gothenburg, as previously described^3^. All Simoa assays were performed on the Simoa HD-X (Quanterix).

The new plasma p-tau231 Simoa assay has been previously described and validated^2^. Briefly, monoclonal mouse antibodies were generated using a synthetic peptide (K224KVAVVR(pT)PPKSPSSAK240C) as a KLH-coupled antigen, numbered according to full-length tau-441 phosphorylated on Thr231. Candidate hybridomas were selected on brain extracts of AD and control brain tissue. The final cloned and purified monoclonal antibody (ADx253) was characterized on synthetic peptides, spanning amino acids Thr217 to Ser241 of full-length tau, for its affinity, its phospho-specificity, using both phosphorylated and nonphosphorylated peptides, and its preferred selectivity in which position 232 was replaced by a Pip, to simulate *cis*-selectivity of ADx253. A biotin-conjugated, amino-terminal, anti-tau mouse monoclonal antibody was used for detection (MAB2241, no. 806502, BioLegend). Full-length recombinant tau-441 phosphorylated in vitro by glycogen synthase kinase 3β was used as the calibrator.

Eli Lilly and Company provided the measurements of the previously published in-house assay for CSF and plasma p-tau217 (ref. ^26^) using the Meso Scale Discovery platform (MSD). This assay uses a streptavidin small spot plate (MSD, L45SA) and customized p-tau217-specific biotinylated monoclonal capture and sulfo-tagged N-terminal tau detection antibodies. The lower limit of quantification of the assay is defined as 0.04 pg ml^−1^ using a customized, synthetic tau dipeptide, standard phosphorylated specifically at Thr217 of the full-length (2N4R) tau protein (synthesized by CPC Scientific). The dipeptide standard contains the epitope of the capture antibody, a poly(ethylene glycol) polymer linker, and the epitope of the detector antibody. The standard was verified to be >95% pure by high-performance liquid chromatography and identity was confirmed by mass spectrometry.

### [^18^F]Flutemetamol PET acquisition and quantification

Participants underwent [^18^F]flutemetamol (amyloid) PET scans after a cranial computed tomography scan for attenuation correction on a Biograph mCT scanner (Siemens Healthcare) at Hospital Clinic, Barcelona. Participants received an intravenous bolus dose of 185 MBq (range 104.25–218.3 MBq, mean ± s.d.: 191.75 ± 14.04) and, 90 min post-injection, PET data were acquired for 20 min (4 frames of 5 min each, mean ± s.d.: 90.15 ± 7.36 min). PET images were reconstructed in 4 frames × 5 min using the three-dimensional Ordered Subset Expectation Maximization algorithm by incorporating time of flight and point spread function modeling.

Amyloid PET processing was performed after a validated Centiloid pipeline^27^ using SPM12 (ref. ^18^). Centiloid values were calculated from the mean values of the standard Centiloid target region (http://www.gaain.org/centiloid-project) and the whole cerebellum as the reference region using the transformation previously calibrated^18^.

Before using the linear transformation to obtain Centiloid values, standardized uptake value ratio images were obtained from the previous images in the MNI space. These images were included in a voxel-wise linear model as a dependent variable to assess their association with the plasma biomarkers (independent variables) in univariate independent models. In all models, age and sex were included as covariates. These analyses were performed with the SPM12 toolbox. The statistical threshold was set at *P* < 0.001 with a cluster size of *k* > 100.

### Statistical analyses

CSF and plasma biomarkers were tested for normality using visual inspection of histograms. None of the biomarkers, except the CSF and plasma Aβ42/Aβ40 ratio, followed a normal distribution and were therefore log_10_-transformed.

Differences in age, education and cognitive performance (MMSE) between Aβ-positive and Aβ-negative groups were assessed using a Student’s *t*-test, whereas group differences in sex and *APOE* ε4 status frequencies were tested using Pearson’s χ^2^ test. Centiloid values, CSF and plasma biomarker levels were compared with a one-way analysis of covariance (ANCOVA), adjusted for age and sex. Similarly, we compared the levels of plasma biomarkers among AT or CSF/PET Aβ groups with an ANCOVA adjusting for age and sex, followed by Tukey’s corrected, post-hoc, pairwise comparisons. The effect size of group differences was estimated by calculating Cohen’s *d*, in which the dependent variable was the residual of log_10_-transformed plasma biomarkers regressed on age and sex.

We modeled the trajectories of plasma biomarkers as a function of Aβ PET (Centiloids) or CSF Aβ42/40, as proxies of progression throughout the preclinical stage of the AD continuum. To do so, we corrected each plasma biomarker value by age and sex and computed the mean and s.d. of each biomarker in a group of participants with CSF Aβ42/40 >0.1, used here as a reference group, and converted biomarker values to *z*-scores. Next, we applied a robust local weighted regression method (rlowess; ‘smooth’ function in Matlab and a span of 300) in 1,000 bootstrap subsamples of the original sample (‘bootstrp’ function in Matlab). The final model was built as the mean *z*-score for all values of the proxy measurements.

Next, we performed ROC analyses to obtain the AUC for Aβ PET (Aβ PET Centiloids ≥12, early cut-off, or Centiloid ≥30, late cut-off) or CSF-defined (CSF Aβ42/40 <0.071) Aβ burden. DeLong’s test was used to compare AUCs for the different plasma biomarkers. Values for sensitivity and specificity were obtained by using Youden’s index cut-off points or setting a sensitivity of 85%. In addition, we performed ROC analyses categorizing participants by age groups using age 65 years or cohorts’ median age (61.8 years) as a cut-off point to define the two age groups.

Finally, we tested whether plasma biomarkers were associated with longitudinal changes in cognitive performance or in Aβ deposition measured with Aβ PET Centiloids. Cognitive performance was assessed with the PACC, which was calculated in the ALFA study was based on the one proposed by Donohue et al.^28^ and the later proposals by Papp et al.^29^ and Jonaitis et al.^30^. According to these previous works, we included categorical fluency measures, and we dropped the MMSE because of its lack of sensitivity^31^. The PACC was calculated averaging the *z*-scores of the Free and Cued Selective Reminding Test (immediate total recall), the WMS-IV Logical Memory test (delayed recall), the WAIS-IV Coding subtest and the Semantic Fluency test (animals in 1 min). The means and s.d. used to create the *z*-scores of the whole sample were calculated from the subsample of individuals with negative AD biomarkers in CSF (A^−^T^−^). PACC scores at visit 2 were also created using the means and s.d. from baseline. Annualized change in the PACC and Centiloids was computed as the subtraction of PACC scores or Centiloid values at visit 2 minus those at visit 1, divided by the time between the two visits in years (mean time PACC: 3.26 (0.31) years; mean time Centiloids: 3.37 (0.44) years). The association between baseline levels of plasma biomarkers and longitudinal change in cognition or Aβ PET was assessed in a linear regression with the annualized change in PACC scores or Centiloid values as the dependent variable, adjusting for age and sex. Years of education were also included as a covariate in the models with annualized change in PACC scores as the dependent variable.

All tests were two tailed, with a significance level of *α* = 0.05, and we corrected for multiple comparisons applying the false discovery rate (FDR) approach^32^, if not otherwise specified. Statistical analyses were performed in SPSS IBM v.20.0, statistical software and the open-source statistical software R v.4.1.2. Figures were built using R and Matlab (v.2018b).

### Reporting summary

Further information on research design is available in the Nature Research Reporting Summary linked to this article.

## Online content

Any methods, additional references, Nature Research reporting summaries, extended data, supplementary information, acknowledgements, peer review information; details of author contributions and competing interests; and statements of data and code availability are available at 10.1038/s41591-022-01925-w.

## Supplementary information

Supplementary Information Supplementary Figs. 1–4 and Tables 1–10.

Reporting Summary

## Data Availability

Requests for the datasets used in the present study will be promptly reviewed by the corresponding authors and the University of Gothenburg and Barcelonaβeta Brain Research Center (BBRC) to verify whether the request is subject to any intellectual property or confidentiality obligations. Anonymized data can be shared by request from any qualified investigator for the sole purpose of replicating procedures and results presented in the article, provided that data transfer is in agreement with EU legislation. Requests received will be reviewed by the BBRC’s Scientific Committee to verify whether these are subject to any intellectual property or confidentiality obligations and compliance with ethical and data protection standards. The BBRC’s Scientific Committee convenes on a quarterly basis and, once approved, the appropriate data sharing agreements will be implemented.

## Extended data

Extended data are available for this paper at 10.1038/s41591-022-01925-w.

## Code availability

All requests for code used for data analyses and data visualization will be promptly reviewed by the corresponding authors and the University of Gothenburg and BBRC to verify whether the request is subject to any intellectual property, confidentiality or other licensing obligations. If there are no limitations, the corresponding authors will communicate with the requester to share the code.
