# Publisher Correction: Plasma p-tau231 and p-tau217 as state markers of amyloid-β pathology in preclinical Alzheimer’s disease

**DOI:** 10.1038/s41591-022-02037-1

**Published:** 2022-09-13

**Authors:** Marta Milà-Alomà, Nicholas J. Ashton, Mahnaz Shekari, Gemma Salvadó, Paula Ortiz-Romero, Laia Montoliu-Gaya, Andrea L. Benedet, Thomas K. Karikari, Juan Lantero-Rodriguez, Eugeen Vanmechelen, Theresa A. Day, Armand González-Escalante, Gonzalo Sánchez-Benavides, Carolina Minguillon, Karine Fauria, José Luis Molinuevo, Jeffrey L. Dage, Henrik Zetterberg, Juan Domingo Gispert, Marc Suárez-Calvet, Kaj Blennow

**Affiliations:** 1 Barcelonaβeta Brain Research Center (BBRC), Pasqual Maragall Foundation, Barcelona, Spain; 2 IMIM (Hospital del Mar Medical Research Institute), Barcelona, Spain; 3 Centro de Investigación Biomédica en Red de Fragilidad y Envejecimiento Saludable, Instituto de Salud Carlos III, Madrid, Spain; 4 Universitat Pompeu Fabra, Barcelona, Spain; 5 Department of Psychiatry and Neurochemistry, Institute of Neuroscience and Physiology, University of Gothenburg, Mölndal, Sweden; 6 Wallenberg Centre for Molecular and Translational Medicine, University of Gothenburg, Gothenburg, Sweden; 7 King’s College London, Institute of Psychiatry, Psychology & Neuroscience, Maurice Wohl Clinical Neuroscience Institute, London, UK; 8 NIHR Biomedical Research Centre for Mental Health & Biomedical Research Unit for Dementia at South London & Maudsley NHS Foundation, London, UK; 9 Translational Neuroimaging Laboratory, McGill Centre for Studies in Aging, McGill University, Montreal, Quebec Canada; 10 Department of Psychiatry, University of Pittsburgh, Pittsburgh, PA USA; 11 ADx NeuroSciences NV, Ghent, Belgium; 12 Lilly Research Laboratories, Eli Lilly and Company, Indianapolis, IN USA; 13 Stark Neurosciences Research Institute, Indiana University School of Medicine, Indianapolis, IN USA; 14 Clinical Neurochemistry Laboratory, Sahlgrenska University Hospital, Mölndal, Sweden; 15 Department of Neurodegenerative Disease, UCL Institute of Neurology, London, UK; 16 UK Dementia Research Institute at UCL, London, UK; 17 Centro de Investigación Biomédica en Red Bioingeniería, Biomateriales y Nanomedicina, Madrid, Spain; 18 Servei de Neurologia, Hospital del Mar, Barcelona, Spain

**Keywords:** Predictive markers, Alzheimer's disease

Correction to: *Nature Medicine* 10.1038/s41591-022-01925-w, published online 11 August 2022.

In the version of this article initially published, the Acknowledgments text, now reading “M.S.C. receives funding from the European Research Council (ERC) under the European Union’s Horizon 2020 research and innovation program (grant agreement no. 948677), the Instituto de Salud Carlos III (PI19/00155), and from the ERC under the EU’s ‘la Caixa’ Foundation (ID 100010434) and from the EU’s Horizon 2020 research and innovation program under the Marie Skłodowska-Curie grant (no. 847648, LCF/BQ/PR21/11840004),” was incomplete and has now been amended in the HTML and PDF versions of the article.

