# Supplementary material for: Plasma p-tau231 and p-tau217 as state markers of amyloid-β pathology in preclinical Alzheimer’s disease
Source: Nat Med. 2022 Aug 11;28(9):1797–801. doi: 10.1038/s41591-022-01925-w (PMC9499867; doi:10.1038/s41591-022-01925-w)
Supplement: Supplementary file 1 — Supplementary Figs. 1–4 and Tables 1–10. [file 41591_2022_1925_MOESM1_ESM.pdf]

nature portfolio

https://doi.org/10.1038/s41591-022-01925-w

Supplementary information

Plasma p-tau231 and p-tau217 as state markers of amyloid-β pathology in preclinical Alzheimer's disease

In the format provided by the authors and unedited

Supplementary Material

Plasma p-tau231 and p-tau217 as state markers of amyloid-β pathology in preclinical Alzheimer's disease

Table of contents

Supplementary Figures 2
Supplementary Fig. 1. Plasma biomarkers by AT group 2
Supplementary Fig. 2. Correlations between CSF and plasma biomarkers in the whole sample 3
Supplementary Fig. 3. Correlations between CSF and plasma biomarkers in Aβ-negative and Aβ-positive participants........ 4
Supplementary Fig. 4. Association of plasma biomarkers with longitudinal change in Aβ deposition by Aβ status (CSF Aβ42/40) 5
Supplementary Tables 6
Supplementary Table 1. Demographic characteristics and biomarker levels by Aβ status 6
Supplementary Table 2. ROC analyses to discriminate Aβ PET Centiloid 12 or 30. 7
Supplementary Table 3. Demographic characteristics and biomarker levels by age groups 8
Supplementary Table 4. ROC analyses to discriminate Aβ status (defined by CSF Aβ42/40) by age groups.. 9
Supplementary Table 5. Plasma biomarkers accuracy for the discrimination of Aβ status (CSF Aβ42/40)....10
Supplementary Table 6. Plasma biomarkers accuracy for the discrimination of Aβ PET Centiloid 30...........11
Supplementary Table 7. Demographic characteristics and biomarker levels by Aβ status of the participants with available longitudinal data 12
Supplementary Table 8. Association of plasma biomarkers with longitudinal changes in cognition 13
Supplementary Table 9. Association of plasma biomarkers with longitudinal changes in Aβ deposition.......14
Supplementary Table 10. Association of plasma biomarkers with longitudinal changes in Aβ deposition in participants with < 30 Centiloids 15

Supplementary Figures

Supplementary Fig. 1. Plasma biomarkers by AT group

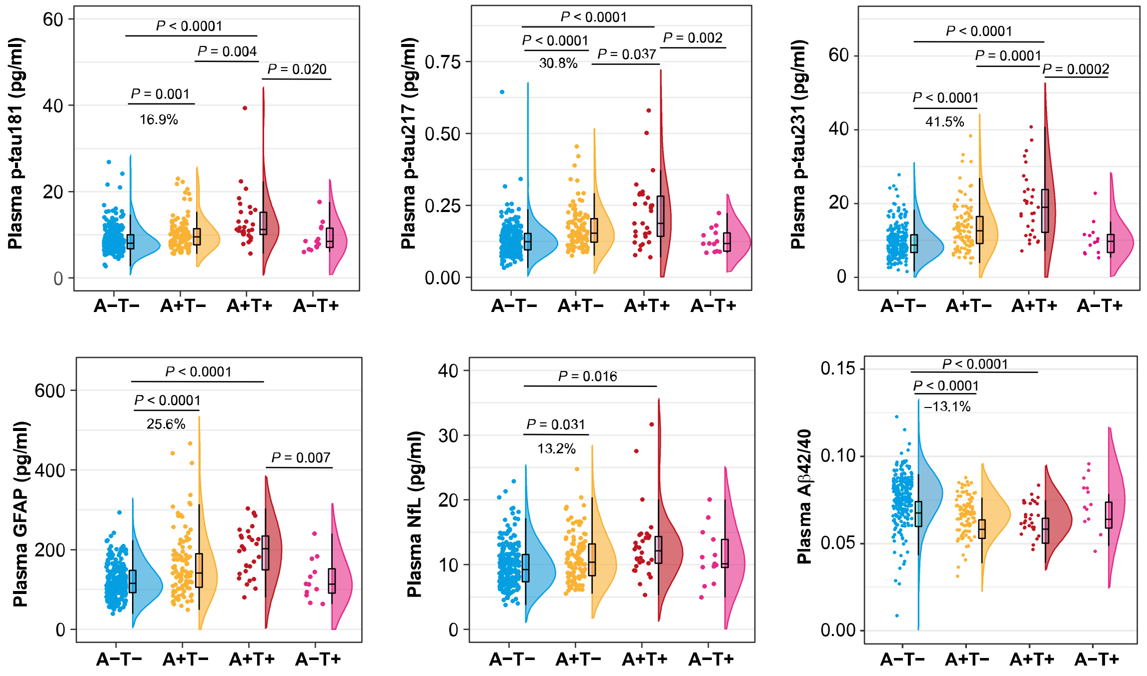

Violin plots comparing plasma biomarkers between AT groups (*n*=397; *n*=249 A−T−, *n*=104 A+T−, *n*=31 A+T+, *n*=13 A−T+). AT groups were defined with CSF Aβ42/40 (A) and CSF Mid(M)-p-tau181 (T). The box plots depict the median (horizontal bar), interquartile range (IQR, hinges), and 1.5 × IQR (whiskers). Group comparisons were computed with a one-way ANCOVA adjusting for age and sex, followed by Tukey-corrected *post hoc* pairwise comparisons. The percentage (%) of change in mean levels of plasma biomarkers in the A+T− group compared to the A−T− one is shown.

Supplementary Fig. 2. Correlations between CSF and plasma biomarkers in the whole sample

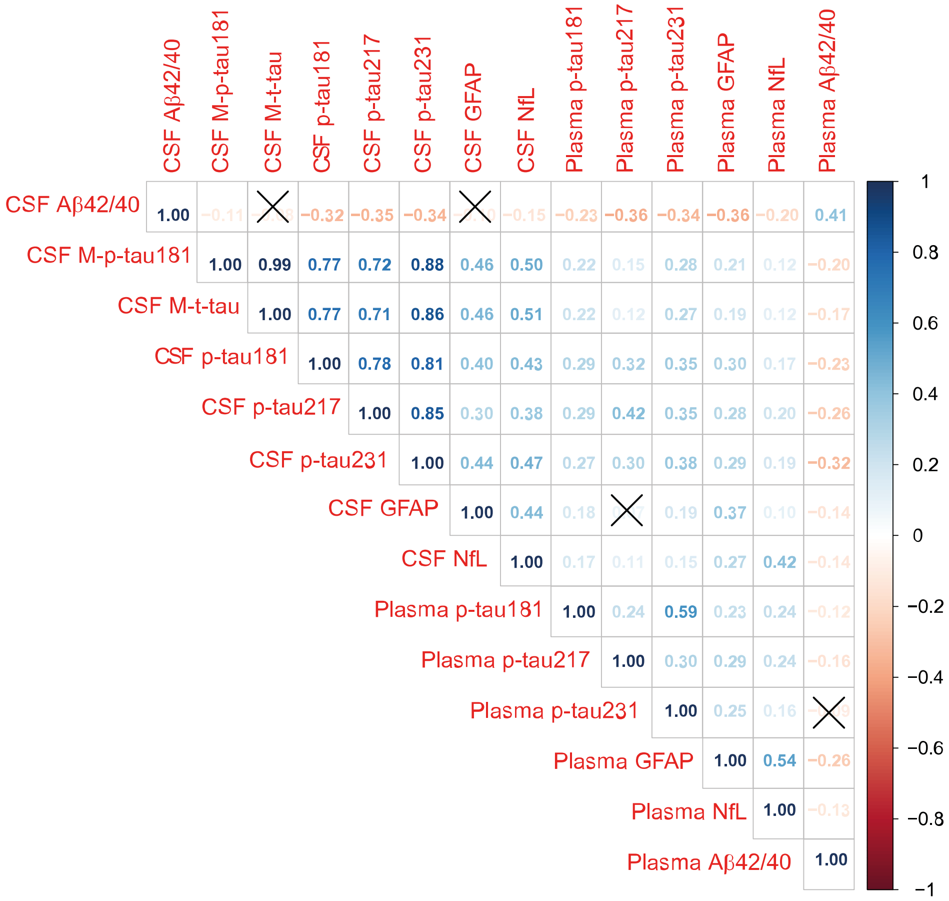

Blue color indicates a positive correlation, and red color a negative correlation. Color intensity is proportional to the Spearman correlation coefficients (*rho*). Crossed cells indicate non-significant correlations.

Supplementary Fig. 3. Correlations between CSF and plasma biomarkers in Aβ-negative and Aβ-positive participants

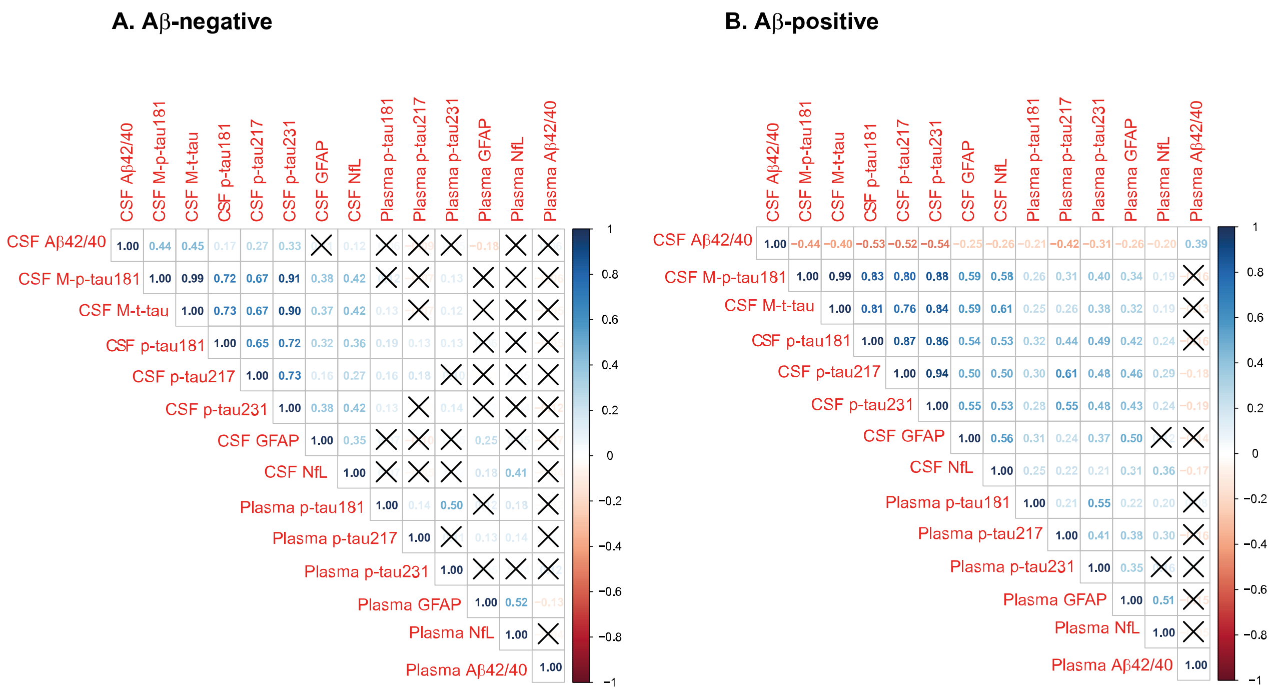

Blue color indicates a positive correlation, and red color a negative correlation. Color intensity is proportional to the Spearman correlation coefficients (*rho*). Crossed cells indicate non-significant correlations. Aβ status was defined by CSF Aβ42/40 (Aβ-positivity: CSF Aβ42/40 < 0.071).

Supplementary Fig. 4. Association of plasma biomarkers with longitudinal change in Aβ deposition by Aβ status (CSF Aβ42/40)

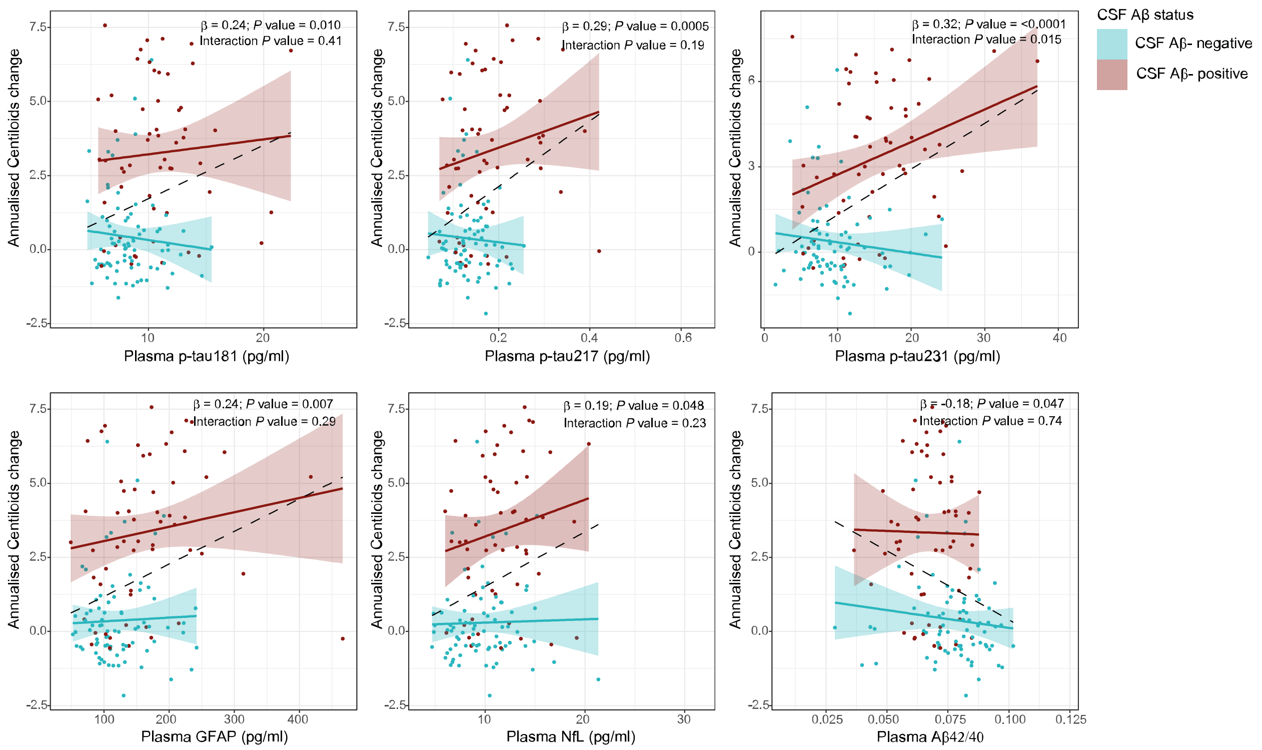

Scatter plots representing the associations of each of the plasma biomarkers with annualised change in Aβ PET Centiloids. Each point depicts the value of the plasma biomarker of an individual and the solid lines indicate the regression line for each of the groups. The dashed line indicates the regression line of the whole sample. The error bands denote the 95% CIs. The standardized regression coefficients (β) and *P* values are shown and were computed using a linear regression with the annualised change in Centiloid values as the dependent variable, adjusting by age and sex. All tests were two-sided. Annualised change in Aβ PET Centiloids was computed as the subtraction of Centiloid values at visit 2 minus those at visit 1 divided by the time difference between the two visits in years.

At the nominal level, there was a significant interaction between CSF Aβ status (as defined by CSF Aβ42/40) and plasma p-tau231. We performed a stratified analysis by CSF Aβ status, and we found a significant association of plasma p-tau231 and longitudinal changes in Aβ PET Centiloids in the Aβ-positive group (β = 0.30, *P* = 0.021), but not in the Aβ-negative group (β = –0.13, *P* = 0.20). See Supplementary Table 9 for the detailed analyses, including FDR correction for multiple testing.

Supplementary Tables

Supplementary Table 1. Demographic characteristics and biomarker levels by Aβ status

	All	Aβ-negative (n = 262)	Aβ-positive (n = 135)	*P* value
**Age,** years	61.1 (4.67)	60.6 (4.45)	62.2 (4.91)	0.0007*
**Female,** n (%)	243 (61.2)	162 (61.8)	81 (60.0)	0.75
**Education,** years	13.5 (3.54)	13.7 (3.49)	13.3 (3.64)	0.38
***APOE* ε4 carriers,** n (%)	214 (53.9)	111 (42.4)	103 (76.3)	<0.0001*
**MMSE**	29.2 (0.95)	29.2 (0.93)	29.1 (0.99)	0.90
**Centiloids**	2.71 (16.7)	−4.51 (6.44)	16.8 (21.1)	<0.0001*
**CSF core biomarkers**				
**Aβ42/40**	0.074 (0.019)	0.087 (0.010)	0.051 (0.012)	<0.0001*
**M-p-tau181** (pg/ml)	15.4 (5.84)	14.9 (5.16)	19.6 (10.0)	<0.0001*
**M-t-tau** (pg/ml)	191 (63.8)	182 (57.0)	228 (89.4)	<0.0001*
**Novel CSF biomarkers**				
**p-tau181** (pg/ml)	333 (218)	270 (101)	466 (305)	<0.0001*
**p-tau217** (pg/ml)	6.17 (6.40)	5.73 (1.83)	10.8 (6.92)	<0.0001*
**p-tau231** (pg/ml)	8.13 (6.04)	6.11 (2.24)	12.4 (8.37)	<0.0001*
**GFAP** (pg/ml)	4361 (2163)	4165 (2227)	4859 (2333)	0.020*
**NfL** (pg/ml)	80.8 (25.7)	78.5 (28.2)	89.2 (27.5)	0.001*
**Plasma biomarkers**				
**p-tau181** (pg/ml)	9.56 (3.90)	8.83 (3.21)	11.0 (4.60)	<0.0001*
**p-tau217** (pg/ml)	0.15 (0.072)	0.13 (0.055)	0.18 (0.086)	<0.0001*
**p-tau231** (pg/ml)	12.0 (10.3)	9.62 (4.33)	15.0 (7.49)	<0.0001*
**GFAP** (pg/ml)	139 (62.3)	122 (42.8)	170 (78.5)	<0.0001*
**NfL** (pg/ml)	10.4 (3.71)	9.90 (3.34)	11.6 (4.19)	0.001*
**Aβ42/40**	0.073 (0.014)	0.076 (0.014)	0.066 (0.10)	<0.0001*

Data shown as mean (SD) or n (%), as appropriate. We used *t* test to compare age, education years and MMSE between groups and Pearson's Chi-square test to compare sex and *APOE* ε4 frequencies between groups. Fluid biomarkers levels and Aβ PET Centiloids were compared with a one-way ANCOVA adjusted by age and sex and followed by FDR multiple comparison correction. All significant (*P* value < 0.05) differences in biomarkers survived FDR multiple comparison correction. Aβ status for group definition was based on CSF Aβ42/40 (cut-off for positivity: CSF Aβ42/40 < 0.071). M-p-tau181 and M-t-tau were measured with Elecsys® immunoassays (Roche Diagnostics International Ltd), which target the mid-region (M) domain of tau protein. All tests were two-sided. *Significant values

Aβ, amyloid-β; *APOE*, apolipoprotein E; GFAP, glial fibrillary acidic protein; MMSE, Mini-Mental State Exam; NfL, neurofilament light; p-tau181, tau phosphorylated at threonine 181; p-tau217, tau phosphorylated at threonine 217; p-tau231, tau phosphorylated at threonine 231.

Supplementary Table 2. ROC analyses to discriminate Aβ PET Centiloid 12 or 30

	**Aβ PET Centiloid 12**			**Aβ PET Centiloid 30**		
	**AUC** [95% CI]	***P* value**	**Adj *P* value**	**AUC** [95% CI]	***P* value**	**Adj *P* value**
Base risk factors model (age + sex + *APOE* ε4)	0.760 [0.696 - 0.823]			0.825 [0.758 - 0.892]		
vs plasma p-tau181	0.734 [0.661 - 0.807][1]	0.61	0.88	0.763 [0.669 - 0.856]	0.27	0.70
vs plasma p-tau217	0.792 [0.722 - 0.862][2]	0.49	0.88	0.854 [0.767 - 0.942]	0.58	0.70
vs plasma p-tau231	0.742 [0.666 - 0.819][1]	0.72	0.88	0.783 [0.674 - 0.891]	0.47	0.70
vs plasma GFAP	0.752 [0.673 - 0.831][3]	0.88	0.88	0.820 [0.720 - 0.920]	0.91	0.91
vs plasma NfL	0.615 [0.529 - 0.701][4]	0.003*	0.015*	0.726 [0.625 - 0.828]	0.039*	0.24
vs plasma Aβ42/40	0.766 [0.706 - 0.827]	0.86	0.88	0.792 [0.725 - 0.859]	0.52	0.70
Base risk factors model (age + sex + *APOE* ε4)	0.760 [0.696 - 0.823]			0.825 [0.758 - 0.892]		
vs plasma p-tau181 + risk factors	0.805 [0.743 - 0.867]	0.030*	0.052	0.864 [0.801 - 0.927]	0.088	0.11
vs plasma p-tau217 + risk factors	0.851 [0.800 - 0.903][5]	0.001*	0.006*	0.925 [0.868 - 0.981]	0.001*	0.008*
vs plasma p-tau231 + risk factors	0.818 [0.758 - 0.877]	0.035*	0.052	0.884 [0.811 - 0.957]	0.039*	0.078
vs plasma GFAP + risk factors	0.810 [0.749 - 0.871]	0.051	0.062	0.875 [0.803 - 0.946]	0.066	0.099
vs plasma NfL + risk factors	0.755 [0.687 - 0.824][6]	0.96	0.96	0.843 [0.766 - 0.920]	0.29	0.29
vs plasma Aβ42/40 + risk factors	0.830 [0.780 - 0.880]	0.002*	0.006*	0.878 [0.827 - 0.930]	0.003*	0.008*

ROC analyses for the discrimination between individuals with Aβ PET Centiloid ≥ 12 or 30 from those with Centiloids < 12 or 30, respectively, for a subset of participants with available Aβ PET data (n = 339). In a first model, we compared each plasma biomarker alone with the base risk factors model (age, sex and *APOE* ε4 status), and in a second model we combined each plasma biomarker with the risk factors. AUCs differences were tested using two-sided DeLong tests followed by FDR multiple comparison correction. *P* values refer to the comparisons with the base risk factors model. *Significant values compared to the base risk factors model.
Plasma biomarker models were also compared between them:

[1]$P < 0.05$ vs plasma NfL
[2]$P < 0.001$ vs plasma NfL
[3]$P < 0.01$ vs plasma NfL
[4]$P < 0.05$ vs plasma Aβ42/40
[5]$P < 0.01$ vs plasma NfL + risk factors
[6]$P < 0.05$ vs plasma Aβ42/40 + risk factors

Aβ, amyloid-β; *APOE*, apolipoprotein E; AUC, area under the curve; CI, confidence interval; GFAP, glial fibrillary acidic protein; NfL, neurofilament light; p-tau181, tau phosphorylated at threonine 181; p-tau217, tau phosphorylated at threonine 217; p-tau231, tau phosphorylated at threonine 231.

Supplementary Table 3. Demographic characteristics and biomarker levels by age groups

	**Younger half** (≤61.8yo; n = 198)	**Older half** (>61.8yo; n = 199)	***P* value**	**Younger group** (≤65yo; n = 309)	**Older group** (>65yo; n = 88)	***P* value**
**Age,** years	57.2 (2.89)	65.0 (2.15)	<0.0001*	59.5 (3.83)	67.0 (1.52)	<0.0001*
**Female,** n (%)	130 (65.7)	113 (56.8)	0.070	193 (62.5)	50 (56.8)	0.40
**Education,** years	13.8 (3.40)	13.3 (3.67)	0.15	13.7 (3.52)	12.9 (3.56)	0.051
***APOE* ε4 carriers,** n (%)	113 (57.1)	101 (50.8)	0.21	172 (55.7)	42 (47.7)	0.23
**MMSE**	29.3 (0.88)	29.0 (1.00)	0.005*	29.2 (0.89)	28.9 (1.08)	0.003*
**Centiloids**	−1.02 (10.5)	6.55 (20.6)	0.0002*	0.051 (13.4)	12.2 (22.9)	<0.0001*
**CSF core biomarkers**						
**Aβ42/40**	0.078 (0.019)	0.072 (0.020)	0.0007*	0.077 (0.019)	0.066 (0.020)	<0.0001*
**M-p-tau181** (pg/ml)	15.1 (6.02)	17.4 (8.71)	0.002*	15.6 (6.58)	18.6 (10.0)	0.0006*
**M-t-tau** (pg/ml)	187 (62.2)	208 (81.1)	0.003*	192 (67.3)	218 (87.6)	0.002*
**Novel CSF biomarkers**						
**p-tau181** (pg/ml)	296 (155)	378 (258)	0.0001*	314 (182)	419 (296)	<0.0001*
**p-tau217** (pg/ml)	6.64 (3.38)	8.25 (5.97)	0.001*	6.90 (4.01)	9.37 (6.93)	<0.0001*
**p-tau231** (pg/ml)	7.22 (4.48)	9.35 (7.04)	0.0003*	7.64 (5.35)	10.3 (7.48)	0.0001*
**GFAP** (pg/ml)	3944 (2044)	4860 (2421)	<0.0001*	4147 (2131)	5292 (2575)	<0.0001*
**NfL** (pg/ml)	72.7 (22.7)	93.0 (33.4)	<0.0001*	77.5 (24.0)	102 (40.9)	<0.0001*
**Plasma biomarkers**						
**p-tau181** (pg/ml)	8.81 (3.01)	10.3 (4.47)	0.0002*	9.25 (3.74)	10.6 (4.15)	0.005*
**p-tau217** (pg/ml)	0.15 (0.069)	0.15 (0.074)	0.66	0.14 (0.066)	0.16 (0.088)	0.059
**p-tau231** (pg/ml)	10.7 (5.24)	12.3 (6.87)	0.027*	11.1 (5.64)	12.8 (7.55)	0.021*
**GFAP** (pg/ml)	123 (50.3)	154 (68.6)	<0.0001*	131 (59.0)	167 (64.3)	<0.0001*
**NfL** (pg/ml)	9.41 (3.40)	11.5 (3.76)	<0.0001*	9.92 (3.44)	12.4 (4.07)	<0.0001*
**Aβ42/40**	0.074 (0.014)	0.071 (0.013)	0.12	0.074 (0.014)	0.069 (0.013)	0.004*

Data shown as mean (SD) or n (%), as appropriate. Participants were stratified by age based on the cohort's median age (61.8 years old) or 65 years old. We used *t* test to compare age, education years and MMSE between groups and Pearson's Chi-square test to compare sex and *APOE* ε4 frequencies between groups. Fluid biomarkers levels and Aβ PET Centiloids were compared with a one-way ANCOVA adjusted by sex and followed by false discovery rate (FDR) multiple comparison correction. All significant (*P* value < 0.05) differences in biomarkers survived FDR multiple comparison correction. M-p-tau181 and M-t-tau were measured with Elecsys® immunoassays (Roche Diagnostics International Ltd), which target the mid-region (M) domain of tau protein. All tests were two-sided. *Significant values.

Aβ, amyloid-β; *APOE*, apolipoprotein E; GFAP, glial fibrillary acidic protein; MMSE, Mini-Mental State Exam; NfL, neurofilament light; p-tau181, tau phosphorylated at threonine 181; p-tau217, tau phosphorylated at threonine 217; p-tau231, tau phosphorylated at threonine 231; yo, years old.

Supplementary Table 4. ROC analyses to discriminate Aβ status (defined by CSF Aβ42/40) by age groups

	**Younger half** (≤61.8yo; n=198)			**Older half** (>61.8yo; n=199)		
	**AUC** [95% CI]	***P* value**	**Adj *P* value**	**AUC** [95% CI]	***P* value**	**Adj *P* value**
Base risk factors model (age + sex + *APOE* ε4)	0.731 [0.659 - 0.802]			0.716 [0.643 - 0.789]		
vs plasma p-tau181	0.657 [0.570 - 0.743]	0.19	0.33	0.666 [0.589 - 0.744]	0.28	0.76
vs plasma p-tau217	0.655 [0.566 - 0.744]	0.22	0.33	0.758 [0.691 - 0.826][1]	0.38	0.76
vs plasma p-tau231	0.758 [0.681 - 0.835]	0.69	0.69	0.721 [0.648 - 0.794][2]	0.91	0.91
vs plasma GFAP	0.632 [0.542 - 0.723]	0.057	0.17	0.728 [0.652 - 0.805][1]	0.72	0.86
vs plasma NfL	0.608 [0.518 - 0.698]	0.039*	0.17	0.593 [0.512 - 0.675][3]	0.019*	0.11
vs plasma Aβ42/40	0.756 [0.684 - 0.828]	0.60	0.69	0.739 [0.671 - 0.808]	0.60	0.86
Base risk factors model (age + sex + *APOE* ε4)	0.731 [0.659 - 0.802]			0.716 [0.643 - 0.789]		
vs plasma p-tau181 + risk factors	0.793 [0.725 - 0.861]	0.041*	0.10	0.755 [0.687 - 0.823]	0.19	0.23
vs plasma p-tau217 + risk factors	0.789 [0.723 - 0.855]	0.070	0.12	0.823 [0.764 - 0.881]	0.0008*	0.005*
vs plasma p-tau231 + risk factors	0.847 [0.791 - 0.903]	0.0006*	0.003*	0.793 [0.730 - 0.857]	0.014*	0.021*
vs plasma GFAP + risk factors	0.783 [0.716 - 0.850]	0.13	0.15	0.792 [0.726 - 0.858]	0.011*	0.021*
vs plasma NfL + risk factors	0.774 [0.709 - 0.840]	0.15	0.15	0.737 [0.664 - 0.809]	0.27	0.27
vs plasma Aβ42/40 + risk factors	0.828 [0.767 - 0.888]	0.0009*	0.003*	0.789 [0.727 - 0.852]	0.004*	0.013*

ROC analyses for the discrimination between Aβ-positive (A+) and Aβ-negative individuals (A–), as defined by the CSF Aβ42/40 ratio (cut-off for positivity: CSF Aβ42/40 < 0.071). Participants were stratified by age based on the cohort median age (61.8 years old) into two halves: a younger and an older group. In a first model, we compared each plasma biomarker with the base risk factors model (age, sex and *APOE* ε4 status), and in a second model we combined each plasma biomarker with the risk factors. *P* values refer to the comparisons with the base risk factors model. AUCs differences were tested using two-sided DeLong test followed by FDR multiple comparison correction. Adjusted and non-adjusted P values are shown. *Significant values compared to the base risk factors model.
Biomarker models were also compared between them:

[1]*P* < 0.01 *vs* plasma NfL
[2]*P* < 0.05 *vs* plasma NfL
[3]*P* < 0.05 *vs* plasma Aβ42/40

Aβ, amyloid-β; APOE, apolipoprotein E; AUC, area under the curve; CI, confidence interval; GFAP, glial fibrillary acidic protein; NfL, neurofilament light; p-tau181, tau phosphorylated at threonine 181; p-tau217, tau phosphorylated at threonine 217; p-tau231, tau phosphorylated at threonine 231; yo, years old.

Supplementary Table 5. Plasma biomarkers accuracy for the discrimination of Aβ status (CSF Aβ42/40)

	Threshold	Sensitivity	Specificity	Youden's Index
**Approach: Youden's Index**				
Plasma p-tau181	9.27	61.8	69.1	30.9
Plasma p-tau217	0.17	48.2	83.4	31.6
Plasma p-tau231	12.3	59.3	80.0	39.3
Plasma GFAP	170	44.7	86.4	31.0
Plasma NfL	11.7	41.7	77. 8	19.4
Plasma Aβ42/40	0.07	74.1	67.3	41.4
Plasma p-tau181 + risk factors	0.30	80.2	60.7	40.9
Plasma p-tau217 + risk factors	0.43	63.7	83.0	46.7
Plasma p-tau231 + risk factors	0.35	72.6	75.0	47.6
Plasma GFAP + risk factors	0.32	78.0	68.3	46.3
Plasma NfL + risk factors	0.44	57.6	78.2	35.8
Plasma Aβ42/40 + risk factors	0.30	84.4	62.7	47.1
**Approach: 85% Sensitivity**				
Plasma p-tau181	7.24	84.7	36.1	
Plasma p-tau217	0.11	85.2	34.0	
Plasma p-tau231	7.89	85.2	41.5	
Plasma GFAP	95.8	84.9	30.1	
Plasma NfL	7.86	84.9	31.0	
Plasma Aβ42/40	0.08	85.2	51.4	
Plasma p-tau181 + risk factors	0.26	84.7	54.4	
Plasma p-tau217 + risk factors	0.23	85.2	53.7	
Plasma p-tau231 + risk factors	0.24	85.2	60.0	
Plasma GFAP + risk factors	0.26	84.9	56.2	
Plasma NfL + risk factors	0.23	84.9	48.4	
Plasma Aβ42/40 + risk factors	0.28	85.2	59.5	

Values for sensitivity and specificity were obtained by using Youden's index cut-off points or setting sensitivity at 85% for the discrimination of Aβ status (cut-off for positivity: CSF Aβ42/40 < 0.071). Risk factors comprise age, sex and *APOE* ε4 status.

Aβ, amyloid-β; *APOE*, apolipoprotein E; GFAP, glial fibrillary acidic protein; NfL, neurofilament light; p-tau181, tau phosphorylated at threonine 181; p-tau217, tau phosphorylated at threonine 217; p-tau231, tau phosphorylated at threonine 231.

Supplementary Table 6. Plasma biomarkers accuracy for the discrimination of Aβ PET Centiloid 30

	Threshold	Sensitivity	Specificity	Youden's Index
**Approach: Youden's Index**				
Plasma p-tau181	9.62	83.3	66.0	49.3
Plasma p-tau217	0.20	73.1	90.0	63.1
Plasma p-tau231	17.2	65.4	89.1	54.5
Plasma GFAP	203	69.2	91.7	60.9
Plasma NfL	11.7	68.0	73.6	41.6
Plasma Aβ42/40	0.07	88.5	59.6	48.0
Plasma p-tau181 + risk factors	0.10	83.3	81.5	64.9
Plasma p-tau217 + risk factors	0.08	92.3	84.2	76.9
Plasma p-tau231 + risk factors	0.08	84.6	78.9	63.5
Plasma GFAP + risk factors	0.06	92.3	73.4	65.7
Plasma NfL + risk factors	0.11	72.0	83.2	55.2
Plasma Aβ42/40 + risk factors	0.08	88.5	76.4	64.8
**Approach: 85% Sensitivity**				
Plasma p-tau181	9.62	83.3	66.0	
Plasma p-tau217	0.15	84.6	68.8	
Plasma p-tau231	7.91	84.6	34.5	
Plasma GFAP	140	84.6	64.1	
Plasma NfL	9.45	84.0	47.5	
Plasma Aβ42/40	0.07	84.6	62.5	
Plasma p-tau181 + risk factors	0.10	83.3	81.5	
Plasma p-tau217 + risk factors	0.13	84.6	88.8	
Plasma p-tau231 + risk factors	0.08	84.6	78.9	
Plasma GFAP + risk factors	0.07	84.6	77.1	
Plasma NfL + risk factors	0.05	84.0	63.4	
Plasma Aβ42/40 + risk factors	0.09	84.6	79.6	

Values for sensitivity and specificity were obtained by using Youden's index cut-off points or setting sensitivity at 85% to detect Aβ PET Centiloids ≥ 30. Risk factors comprise age, sex and *APOE* ε4 status.

Aβ, amyloid-β; *APOE*, apolipoprotein E; GFAP, glial fibrillary acidic protein; NfL, neurofilament light; p-tau181, tau phosphorylated at threonine 181; p-tau217, tau phosphorylated at threonine 217; p-tau231, tau phosphorylated at threonine 231.

Supplementary Table 7. Demographic characteristics and biomarker levels by Aβ status of the participants with available longitudinal data

	All	Aβ-negative (n = 142)	Aβ-positive (n = 72)	*P* value
**Age, years**	60.7 (4.94)	60.1 (4.65)	61.9 (5.32)	0.013*
**Female,** n (%)	137 (64.0)	91.0 (64.1)	46 (63.9)	1.00
**Education,** years	13.5 (3.60)	13.6 (3.51)	13.3 (3.80)	0.59
***APOE* ε4 carriers,** n (%)	102 (47.7)	50.0 (35.2)	52 (72.2)	<0.0001*
**MMSE**	29.2 (0.99)	29.1 (1.01)	29.3 (0.96)	0.25
**Centiloids**	3.73 (17.3)	−3.62 (6.32)	18.0 (22.3)	<0.0001*
**CSF core biomarkers**				
**Aβ42/40**	0.076 (0.021)	0.088 (0.010)	0.051 (0.012)	<0.0001*
**M-p-tau181** (pg/ml)	16.9 (8.59)	15.1 (5.45)	20.3 (12.01)	0.0002*
**M-t-tau** (pg/ml)	204 (80.0)	189 (59.3)	236 (104)	0.0003*
**Novel CSF biomarkers**				
**p-tau181** (pg/ml)	344 (218)	279 (96.4)	474 (317)	<0.0001*
**p-tau217** (pg/ml)	7.84 (5.08)	6.15 (1.76)	11.2 (7.38)	<0.0001*
**p-tau 231** (pg/ml)	8.57 (6.22)	6.41 (2.35)	12.9 (8.83)	<0.0001*
**GFAP** (pg/ml)	4462 (2454)	4136 (2424)	5100 (2400)	0.028*
**NfL** (pg/ml)	82.3 (31.2)	76.1 (25.3)	94.7 (37.5)	0.0004*
**Plasma biomarkers**				
**p-tau181** (pg/ml)	9.83 (3.81)	9.05 (3.42)	11.4 (4.11)	<0.0001*
**p-tau217** (pg/ml)	0.15 (0.078)	0.14 (0.063)	0.19 (0.093)	<0.0001*
**p-tau231** (pg/ml)	11.9 (6.53)	9.72 (4.59)	16.2 (7.59)	<0.0001*
**GFAP** (pg/ml)	137 (62.8)	120 (43.1)	169 (80.8)	<0.0001*
**NfL** (pg/ml)	10.5 (3.79)	9.84 (3.30)	11.9 (4.32)	0.002*
**Aβ42/40**	0.073 (0.013)	0.076 (0.013)	0.066 (0.011)	<0.0001*
**Longitudinal changes**				
**Annualised PACC change**	0.030 (0.12)	0.045 (0.12)	0.001 (0.12)	0.012*
**Annualised Centiloid change****	1.65 (2.43)	0.37 (1.48)	3.34 (2.40)	<0.0001*

Data shown as mean (SD) or n (%), as appropriate. We used *t* test to compare age, education years and MMSE between groups and Pearson's Chi-square test to compare sex and *APOE* ε4 frequencies between groups. Fluid biomarkers levels, Aβ PET Centiloids and annualised Centiloids and PACC scores change were compared with a one-way ANCOVA adjusted by age and sex and followed by false discovery rate (FDR) multiple comparison correction. All significant (*P* value < 0.05) differences in biomarkers survived FDR multiple comparison correction. Aβ status for group definition was based on CSF Aβ42/40 (cut-off for positivity: CSF Aβ42/40 < 0.071). M-p-tau181 and M-t-tau were measured with Elecsys® immunoassays (Roche Diagnostics International Ltd), which target the mid-region (M) domain of tau protein. All tests were two-sided.
*Significant values
**Longitudinal Centiloids data was available for a subset of 145 participants.

Aβ, amyloid-β; *APOE*, apolipoprotein E; GFAP, glial fibrillary acidic protein; MMSE, Mini-Mental State Exam; NfL, neurofilament light; PACC, Preclinical Alzheimer Cognitive Composite; p-tau181, tau phosphorylated at threonine 181; p-tau217, tau phosphorylated at threonine 217; p-tau231, tau phosphorylated at threonine 231.

Supplementary Table 8. Association of plasma biomarkers with longitudinal changes in cognition

	**Main effect of plasma biomarkers on Annualised PACC change**				**'Plasma biomarker × Aβ status' Interaction**	
	**β** (95% CI)	***t* statistic**	***P* value**	**Adj *P* value**	***P* value**	**Adj *P* value**
**Plasma p-tau181**	–0.21 (–0.35 - –0.070)	–2.97	0.003*	0.020*	0.26	0.53
**Plasma p-tau217**	–0.063 (–0.20 - 0.072)	–0.92	0.36	0.57	0.48	0.72
**Plasma p-tau231**	–0.099 (–0.23 - 0.028)	–1.54	0.13	0.38	0.027*	0.16
**Plasma GFAP**	–0.017 (–0.17 - 0.14)	–0.22	0.83	0.83	0.77	0.92
**Plasma NfL**	–0.067 (–0.22 - 0.083)	–0.88	0.38	0.57	0.12	0.35
**Plasma Aβ42/40**	0.049 (–0.11 - 0.20)	0.62	0.54	0.64	0.94	0.94

The association between each plasma biomarker and cognitive change was assessed in a linear regression with the annualised change in PACC scores as dependent variable, adjusting by age, sex and education years. Additionally, we tested the interaction terms between each biomarker and CSF Aβ status. At the nominal level, there was a significant interaction between CSF Aβ status and plasma p-tau231. Thus, we performed an analysis stratifying by Aβ status and we found a significant association of plasma p-tau231 with PACC score longitudinal changes only in the Aβ-positive group ($\beta = -0.27$, $P = 0.023$), but not in the Aβ-negative group ($\beta = 0.054$; $P = 0.51$). All tests were two-sided and *P* values were adjusted for multiple comparisons using FDR approach.
*Significant values

Aβ, amyloid-β; GFAP, glial fibrillary acidic protein; NfL, neurofilament light; PACC, Preclinical Alzheimer Cognitive Composite; p-tau181, tau phosphorylated at threonine 181; p-tau217, tau phosphorylated at threonine 217; p-tau231, tau phosphorylated at threonine 231.

Supplementary Table 9. Association of plasma biomarkers with longitudinal changes in Aβ deposition

	Main effect of plasma biomarkers on Annualised Centiloid change				'Plasma biomarker × Aβ status' Interaction	
	**β** (95% CI)	***t* statistic**	***P* value**	**Adj *P* value**	***P* value**	**Adj *P* value**
**Plasma p-tau181**	0.24 (0.057 - 0.42)	2.60	0.010*	0.016*	0.41	0.49
**Plasma p-tau217**	0.29 (0.13 - 0.45)	3.56	0.0005*	0.002*	0.19	0.43
**Plasma p-tau231**	0.32 (0.18 - 0.47)	4.42	<0.0001*	0.0001*	0.015*	0.089
**Plasma GFAP**	0.24 (0.067 - 0.42)	2.74	0.007*	0.014*	0.29	0.43
**Plasma NfL**	0.19 (0.002 - 0.38)	2.00	0.048*	0.048*	0.23	0.43
**Plasma Aβ42/40**	–0.18 (–0.36 - –0.003)	–2.01	0.047*	0.048*	0.74	0.74

The association between each plasma biomarker and changes in Aβ deposition was assessed in a linear regression with the annualised change in Aβ PET Centiloid values as dependent variable, adjusting by age and sex. Additionally, we tested the interaction terms between each biomarker and Aβ status (as defined by CSF Aβ42/40). At the nominal level, there was a significant interaction between Aβ status and plasma p-tau231. Thus, we performed an analysis stratifying by Aβ status and we found a significant association of plasma p-tau231 with Aβ PET Centiloid longitudinal changes only in the Aβ-positive group ($\beta = 0.30$, $P = 0.021$) but not in the Aβ-negative group ($\beta = -0.13$, $P = 0.20$). All tests were two-sided and *P* values were adjusted for multiple comparisons using FDR approach. *Significant values

Aβ, amyloid-β; GFAP, glial fibrillary acidic protein; NfL, neurofilament light; p-tau181, tau phosphorylated at threonine 181; p-tau217, tau phosphorylated at threonine 217; p-tau231, tau phosphorylated at threonine 231.

Supplementary Table 10. Association of plasma biomarkers with longitudinal changes in Aβ deposition in participants with < 30 Centiloids

	Main effect of plasma biomarkers on Annualised Centiloid change			
	**β** (95% CI)	***t* statistic**	***P* value**	**Adj *P* value**
**Plasma p-tau181**	0.14 (–0.063 - 0.34)	1.37	0.17	0.17
**Plasma p-tau217**	0.22 (0.046 - 0.40)	2.50	0.014*	0.041*
**Plasma p-tau231**	0.23 (0.064 - 0.40)	2.73	0.007*	0.041*
**Plasma GFAP**	0.16 (–0.045 - 0.36)	1.54	0.13	0.17
**Plasma NfL**	0.15 (–0.056 - 0.35)	1.44	0.15	0.17
**Plasma Aβ42/40**	–0.17 (–0.37 - 0.033)	–1.65	0.10	0.17

The association between each plasma biomarker and changes in Aβ deposition was assessed in a linear regression with the annualised change in Aβ PET Centiloid values as dependent variable, adjusting by age and sex. All tests were two-sided and *P* values were adjusted for multiple comparisons using FDR approach.
*Significant values

Aβ, amyloid-β; GFAP, glial fibrillary acidic protein; NfL, neurofilament light; p-tau181, tau phosphorylated at threonine 181; p-tau217, tau phosphorylated at threonine 217; p-tau231, tau phosphorylated at threonine 231.
