# Supplementary material for: Plasma p-tau231 and p-tau217 as state markers of amyloid-β pathology in preclinical Alzheimer’s disease
Source: Nat Med. 2022 Aug 11;28(9):1797–801. doi: 10.1038/s41591-022-01925-w (PMC9499867; doi:10.1038/s41591-022-01925-w)
Supplement: Supplementary file 2 — Reporting Summary [file 41591_2022_1925_MOESM2_ESM.pdf]

nature research

Corresponding author(s): Suárez-Calvet; Blennow

Last updated by author(s): Jun 24, 2022

Reporting Summary

Nature Research wishes to improve the reproducibility of the work that we publish. This form provides structure for consistency and transparency in reporting. For further information on Nature Research policies, see our Editorial Policies and the Editorial Policy Checklist.

Statistics

For all statistical analyses, confirm that the following items are present in the figure legend, table legend, main text, or Methods section.

n/a	Confirmed	
☐	☒	The exact sample size (n) for each experimental group/condition, given as a discrete number and unit of measurement
☐	☒	A statement on whether measurements were taken from distinct samples or whether the same sample was measured repeatedly
☐	☒	The statistical test(s) used AND whether they are one- or two-sided<br>*Only common tests should be described solely by name; describe more complex techniques in the Methods section.*
☐	☒	A description of all covariates tested
☐	☒	A description of any assumptions or corrections, such as tests of normality and adjustment for multiple comparisons
☐	☒	A full description of the statistical parameters including central tendency (e.g. means) or other basic estimates (e.g. regression coefficient) AND variation (e.g. standard deviation) or associated estimates of uncertainty (e.g. confidence intervals)
☐	☒	For null hypothesis testing, the test statistic (e.g. F, t, r) with confidence intervals, effect sizes, degrees of freedom and P value noted<br>*Give P values as exact values whenever suitable.*
☒	☐	For Bayesian analysis, information on the choice of priors and Markov chain Monte Carlo settings
☒	☐	For hierarchical and complex designs, identification of the appropriate level for tests and full reporting of outcomes
☐	☒	Estimates of effect sizes (e.g. Cohen's d, Pearson's r), indicating how they were calculated

Our web collection on statistics for biologists contains articles on many of the points above.

Software and code

Policy information about availability of computer code

Data collection	Data is exported to csv files and stored at Barcelonabeta Brain Research Center
Data analysis	Amyloid PET processing and voxel-wise analysis were performed using SPM12. Statistical analyses were performed in SPSS IBM, version 20.0, statistical software and the open-source statistical software R, version 4.1.2. Figures were built using R and Matlab (v2018b).

For manuscripts utilizing custom algorithms or software that are central to the research but not yet described in published literature, software must be made available to editors and reviewers. We strongly encourage code deposition in a community repository (e.g. GitHub). See the Nature Research guidelines for submitting code & software for further information.

Data

Policy information about availability of data

All manuscripts must include a data availability statement. This statement should provide the following information, where applicable:

- Accession codes, unique identifiers, or web links for publicly available datasets
- A list of figures that have associated raw data
- A description of any restrictions on data availability

Requests for the datasets used in this study will be promptly reviewed by the corresponding authors and the University of Gothenburg and Barcelonaβeta Brain Research Center (BBRC) to verify whether the request is subject to any intellectual property or confidentiality obligations. Anonymized data can be shared by request from any qualified investigator for the sole purpose of replicating procedures and results presented in the article, providing data transfer is in agreement with EU legislation. Requests received will be reviewed by the BBRC's Scientific Committee to verify whether these are subject to any intellectual property or confidentiality obligations and compliance with ethical and data protection standards. The BBRC's Scientific Committee convenes on a quarterly basis and once approved, the appropriate data sharing agreements will be implemented.

Field-specific reporting

Please select the one below that is the best fit for your research. If you are not sure, read the appropriate sections before making your selection.

☒ Life sciences ☐ Behavioural & social sciences ☐ Ecological, evolutionary & environmental sciences

For a reference copy of the document with all sections, see nature.com/documents/nr-reporting-summary-flat.pdf

Life sciences study design

All studies must disclose on these points even when the disclosure is negative.

Sample size	This study was performed in the ALFA+ cohort. The ALFA+ study includes 450 participants that were invited to participate based on their specific AD risk profile. In the present study, we included 397 individuals with available biomarkers measurements. All participants with biomarkers measurements were included and no a priori sample size calculation was done. Longitudinal analyses were performed in a subset of participants with available longitudinal cognitive (n = 214) and Aβ PET (n = 145) data.
Data exclusions	Among the 450 participants of ALFA+, we included the 397 with biomarker data.
Replication	We perform different type of analyses to test whether plasma biomarkers were changed in the early stages of Preclinical Alzheimer. We use the AT classification, we define a group of low Aβ burden, we modeled the changes of plasma biomarkers as a function of CSF Aβ42/40 and amyloid PET and we determined the accuracy of these biomarkers to discriminate between Aβ-positive and Aβ-negative cognitively unimpaired individuals. Several pieces of evidence consistently support that plasma p-tau231 and p-tau217 were the biomarkers indicating very early Aβ changes. This is a unicenter study and hence there is no replication in an independent cohort. All data analyses were run multiple times for confirmation of the findings and all replication attempts were successful.
Randomization	This is an observational study and no allocation into experimental groups were performed. Therefore, randomization is not relevant to this study.
Blinding	All biomarkers analyses were performed by researchers that were blinded to the clinical data of the participants.

Reporting for specific materials, systems and methods

We require information from authors about some types of materials, experimental systems and methods used in many studies. Here, indicate whether each material, system or method listed is relevant to your study. If you are not sure if a list item applies to your research, read the appropriate section before selecting a response.

Materials & experimental systems

n/a	Involved in the study	
☐	☒	Antibodies
☒	☐	Eukaryotic cell lines
☒	☐	Palaeontology and archaeology
☒	☐	Animals and other organisms
☐	☒	Human research participants
☐	☒	Clinical data
☒	☐	Dual use research of concern

Methods

n/a	Involved in the study	
☒	☐	ChIP-seq
☒	☐	Flow cytometry
☒	☐	MRI-based neuroimaging

Antibodies

Antibodies used	The novel plasma pTau231 Simoa assay was previously described and validated (Ashton et al. Acta Neuropathologica 2021). Briefly, monoclonal mouse antibodies were generated using a synthetic peptide (K224KVAVVR(pT)PPKSPSSAK240C) as a KLH-coupled antigen, numbered according to full-length tau-441 phosphorylated on threonine 231. Candidate hybridomas were selected on brain extracts of AD and control brain tissue. The final cloned and purified monoclonal antibody (ADx253) was characterized on synthetic peptides spanning amino acids threonine 217 till serine 241 of full-length tau for its affinity, its phospho-specificity using both phosphorylated and non-phosphorylated peptides and its preferred selectivity in which position 232 was replaced by a Pip, to simulate cis-selectivity of ADx253. A biotin-conjugated N-terminal anti-tau mouse monoclonal antibody was used for detection (MAB2241; #806502, BioLegend, CA, USA). Full-length recombinant tau 441 phosphorylated in vitro by glycogen synthase kinase 313 was used as the calibrator. Eli Lilly and Company provided the measurements of the previously published in-house assay for plasma p-tau217 using the Meso Scale Discovery platform (MSD, Rockville, MD, USA). This assay uses a streptavidin small spot plate (MSD, L45SA) and custom p-tau217-specific biotinylated monoclonal capture and sulfo-tagged amino-terminal tau detection antibodies. The lower limit of quantification of the assay is defined as 0.04 pg/ml using a custom synthetic tau dipeptide standard phosphorylated specifically at threonine 217 of the full-length (2N4R) tau protein (synthesized by CPC Scientific). The dipeptide standard contains the epitope of the capture antibody, a polyethylene glycol polymer linker, and the epitope of the detector antibody. The standard was verified to be >95% pure by HPLC and identity was confirmed by mass spectrometry. The rest of the assays measured are reported in the methods section.

Validation	The plasma p-tau231 and p-tau217 assays were previously validated (Ashton et al. Acta Neuropathologica 2021; Thijssen et al. Lancet Neurology 2021).

Human research participants

Policy information about studies involving human research participants

Population characteristics	Detailed information of the participants' characteristics is provided in Supplementary Table 1. In brief, we included 262 individuals that were amyloid-negative and 135 that were amyloid-positive. The mean age was 61.1 (4.67), 61.2% were female and 53.9% APOE-ε4 carriers.
Recruitment	This study was performed in the ALFA+ cohort (ALFA-FPM-0311), a nested longitudinal study from the ALFA (for ALzheimer's and FAmilies) study. The inclusion and exclusion criteria of ALFA+ are described in the methods section. The ALFA study (45-65/FPM2012 study) includes 2,743 middle-aged, cognitively unimpaired individuals (CDR = 0; MMSE ≥ 26; semantic fluency ≥ 12), with a high proportion of AD patients' offspring and APOE-ε4 carriers. The recruitment, design and inclusion/exclusion criteria of ALFA are comprehensively described in Molinuevo et al. Alzheimer's and Dementia 2016.<br>ALFA+ includes participants with a higher risk for AD by design (high prevalence of APOE ε4 carriership and Aβ positivity) and, therefore, it does not represent normal aging in the general population. This is discussed as a limitation.
Ethics oversight	The ALFA+ study (ALFA-FPM-0311) was approved by the Independent Ethics Committee "Parc de Salut Mar", Barcelona, and registered at Clinicaltrials.gov (Identifier: NCT02485730). All participating subjects signed the study's informed consent form that had also been approved by the Independent Ethics Committee "Parc de Salut Mar", Barcelona.

Note that full information on the approval of the study protocol must also be provided in the manuscript.

Clinical data

Policy information about clinical studies

All manuscripts should comply with the ICMJE guidelines for publication of clinical research and a completed CONSORT checklist must be included with all submissions.

Clinical trial registration	NCT02485730
Study protocol	Provision of the clinical protocol will be considered upon request by qualified researchers.
Data collection	Data and sample collection of the baseline visit of the ALFA+ study was conducted at the BarcelonaBeta Brain Research Center in Barcelona (Spain) between October 2016 and December 2019. The follow-up visits were initiated in November 2019 and are currently ongoing, expected to finalise by Q4 2022.
Outcomes	The ALFA+ study has the aim to characterize the biomarkers changes in Preclinical Alzheimer and the primary outcome is the change from preclinical phase of AD to mild cognitive impairment. In this particular study, we tested whether plasma biomarkers (p-tau181, p-tau217, p-tau231, GFAP, NfL, AB42/40): 1. change in the AT and low Aβ burden groups; 2. change as a function of Aβ PET and CSF Aβ42/40; 3. are associated with Aβ PET uptake; 4. discriminate between Aβ-positive and Aβ-negative cognitively unimpaired individuals; 5. are associated with longitudinal changes in cognition and Aβ PET uptake.
